# Epidural anesthesia needle guidance by forward-view endoscopic optical coherence tomography and deep learning

**DOI:** 10.1038/s41598-022-12950-7

**Published:** 2022-05-31

**Authors:** Chen Wang, Paul Calle, Justin C. Reynolds, Sam Ton, Feng Yan, Anthony M. Donaldson, Avery D. Ladymon, Pamela R. Roberts, Alberto J. de Armendi, Kar-ming Fung, Shashank S. Shettar, Chongle Pan, Qinggong Tang

**Affiliations:** 1 Stephenson School of Biomedical Engineering, University of Oklahoma, Norman, OK 73019 USA; 2 School of Computer Science, University of Oklahoma, Norman, OK 73019 USA; 3 Department of Anesthesiology, University of Oklahoma Health Sciences Center, Oklahoma City, OK 73104 USA; 4 Department of Pathology, University of Oklahoma Health Sciences Center, Oklahoma City, OK 73104 USA; 5 Stephenson Cancer Center, University of Oklahoma Health Sciences Center, Oklahoma City, OK 73104 USA; 6 Institute for Biomedical Engineering, Science, and Technology (IBEST), University of Oklahoma, Norman, OK 73019 USA

**Keywords:** Biomedical engineering, Medical imaging, Endoscopy

## Abstract

Epidural anesthesia requires injection of anesthetic into the epidural space in the spine. Accurate placement of the epidural needle is a major challenge. To address this, we developed a forward-view endoscopic optical coherence tomography (OCT) system for real-time imaging of the tissue in front of the needle tip during the puncture. We tested this OCT system in porcine backbones and developed a set of deep learning models to automatically process the imaging data for needle localization. A series of binary classification models were developed to recognize the five layers of the backbone, including fat, interspinous ligament, ligamentum flavum, epidural space, and spinal cord. The classification models provided an average classification accuracy of 96.65%. During puncture, it is important to maintain a safe distance between the needle tip and the dura mater. Regression models were developed to estimate that distance based on the OCT imaging data. Based on the Inception architecture, our models achieved a mean absolute percentage error of 3.05% ± 0.55%. Overall, our results validated the technical feasibility of using this novel imaging strategy to automatically recognize different tissue structures and measure the distances ahead of the needle tip during the epidural needle placement.

## Introduction

Epidural anesthesia has become a well-established anesthetic method widely used in painless delivery^1^, thoracic surgeries^2^, orthopedic surgeries^3^, organ transplantation surgeries^4^, abdominal surgeries^5^, and chronic pain relief^6^. Epidural anesthesia uses an epidural needle to inject the anesthetic medications into the epidural space, which averages 1–6 mm in width and several centimeters in depth behind the skin layer^7^. During the placement of the epidural needle, the epidural needle penetrates subcutaneous fat, supraspinous ligament, interspinous ligament and ligamentum flavum before reaching the epidural space (between flavum and dura mater) to inject the medications^8^. Therefore, accurate positioning of the needle in the epidural space is critical for safe and effective epidural anesthesia.

Inadvertent penetration and damage to neurovascular structures leads to several complications, such as headache, transient paresthesia, and severe epidural hematomas^1^. Puncturing the dura will cause excessive loss of cerebrospinal fluid (CSF) and can damage nerves in the spinal cord^9^. It has been reported that more than 6% of patients have abnormal feelings during the placement of needle, and this has been shown to be a risk factor of persistent paresthesia^10^. Post dural puncture headache (PDPH) is one of the most common complications in epidural anesthesia^11^. It occurs in over 50% of the accidental dural puncture cases^12^. Some researchers reported the PDPH incidence rate for females was two to three times greater than men, and pregnancy could further increase the possibility of PDPH^13^. Besides PDPH, more serious consequences such as spinal cord damage, paralysis or epidural hematoma and the development of an abscess might occur due to inaccurate puncture^14,15^. Moreover, the neurologic injury caused by inadvertent puncture can lead to other symptoms like fever or photophobia^16,17^.

In current clinical practice, accurate placement of the needle relies on the experience of the anesthesiologist^18^. The most common method of detecting the placement of the needle in the epidural space is based on the loss of resistance (LOR)^19^. To test the LOR, the anesthesiologist keeps pressing on the plunger of a syringe filled with saline or air during the inserting the epidural needle^20^. When the needle tip passes through the ligamentum flavum and reaches at the epidural space, there is a sudden decrease of the resistance that can be sensed by anesthesiologists^21^. Nevertheless, this method has been shown to be inaccurate in predicting needle location and actual needle insertion could be further inside the body than the expectation^22^. Up to 10% of patients undergoing epidural anesthesia are not provided with adequate analgesia by using LOR^23,24^. And the LOR technique can fail in up to 53% of the attempts without image guidance in more challenging procedures such as cervical epidural injections^25,26^. Moreover, complications such as pneumocephalus^27^, nerve root compression^28^, subcutaneous emphysema^29^ and venous air embolism^30^ have been shown to be related to the air or liquid injection while using LOR technique. To improve the success rate of epidural puncture and decrease the number of puncture attempts, there is a strong demand for an effective imaging technique to guide the epidural needle insertion.

Currently, imaging modalities, such as ultrasound and fluoroscopy, have been utilized during the needle access^31,32^. However, the complex and articulated encasement of bones allows only a narrow acoustic window for the ultrasound beam^26^. Fluoroscopy does not have soft tissue contrast and, thus, cannot differentiate critical soft tissues (such as blood vessels and nerve roots) that need to be avoided during the needle insertion. Moreover, the limited resolution and contrast in fluoroscopy make it difficult to distinguish different tissue layers in front of the needle tip, especially for the cervical and thoracic epidural anesthesia where the epidural space is as narrow as 1–4 mm^33^. To improve the needle placement accuracy, novel optical imaging systems have been designed and tested. A portable optical epidural needle system based on fiberoptic bundle was designed to identify the epidural space^34^, but there are some limitations for the optical signal interpretation and needle trajectory identification due to the uncertain direction of needle bevel or the surrounding fluid^35^. Additionally, optical spectral analysis has been utilized for tissue differentiation during epidural space identification^36,37^. However, the accuracy of measured spectral results can be compromised by the surrounding tissues and the bleeding during the puncture.

Optical coherence tomography (OCT) is a non-invasive imaging modality that can visualize the cross-sections of tissue samples^38^. At 10–100 times higher resolution (~ 10 µm) than ultrasound and fluoroscopy, OCT can improve the efficacy of tissue imaging^39^. OCT has been integrated with fiber-optic catheters and endoscopes for numerous internal imaging applications^40–43^. Fiber-optic based OCT probe systems have been proposed in epidural anesthesia needle guidance and provided promising results in identifying epidural space in pig models^44,45^. In the previous study, our group has also reported a forward-imaging endoscopic OCT needle device for real-time epidural anesthesia placement guidance and demonstrated its feasibility in piglets in vivo^26^. By fitting the OCT needle inside the hollow bore of the epidural needle, no additional invasiveness is introduced from the OCT endoscope. The high scanning speed of OCT system allows real-time imaging of the tissue OCT images in front of the needle. The tissues in front of the needle tip can be recognized based on the distinct OCT imaging features of the different tissues.

Convolutional neural networks (CNN) has been widely used for classification of medical images^46,47^ and have been applied for OCT images in macular, retina and esophageal related research for automatic tissue segmentation^48–50^. To help improve the efficiency of tissue recognition, herein we proposed to use CNN to classify and recognize different epidural tissue types automatically. In this study, we developed a computer-aided diagnosis (CAD) system based on CNN to automatically locate the epidural needle tip based on the forward-view OCT images. To the best of our knowledge, this is the first attempt to combine forward-view OCT system with CNN for guiding the epidural anesthesia procedure. Five epidural layers (fat, interspinous ligament, ligamentum flavum, epidural space and spinal cord) were imaged to train and test the CNN classifiers based on Inception^51^, Residual Network 50 (ResNet50)^52^ and Xception^53^. After the needle tip arrives the epidural space, the OCT images can then be used to estimate the distance of the needle tip from the dura mater to avoid spinal cord damage. We trained and tested regression models based on Inception, ResNet50 and Xception using OCT images with manually labeled distances. The Inception model achieved the best performance with a mean absolute percentage error of 3.05% ± 0.55%. These results demonstrated the feasibility of this novel imaging strategy for guiding the epidural anesthesia needle placement.

## Results

### OCT images of five epidural layer categories

**Figure 1:**
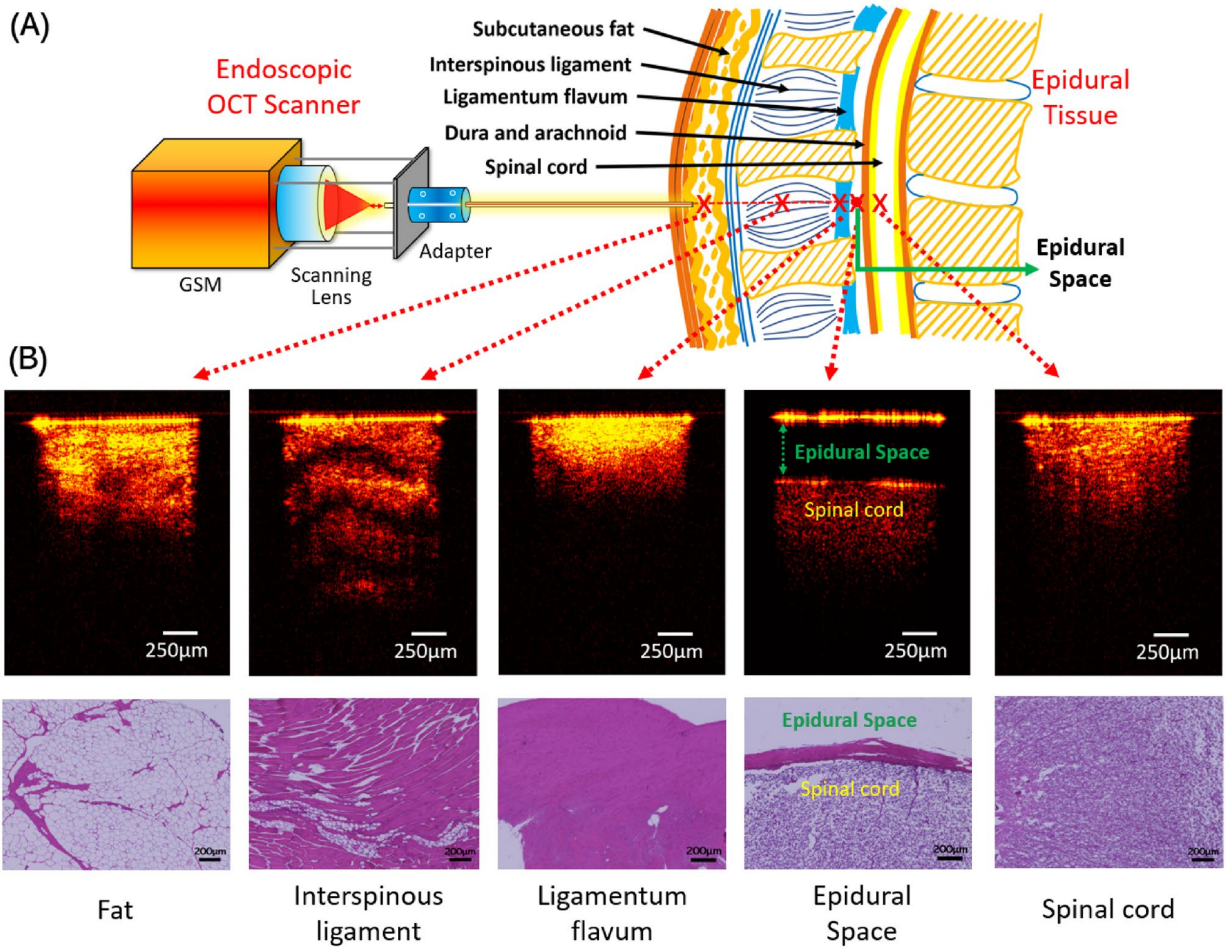
(**A**) Endoscopic OCT scanner setup and the representative OCT images of five epidural tissue layer categories. (**B**) Histology results of different tissue layers.

The schematic of the experiment using our endoscopic OCT system was shown in Fig. 1A. Cross-sectional 2D OCT image examples of fat, interspinous ligament, ligamentum flavum, epidural space and spinal cord were shown in Fig. 1B. Because of the gap between needle tip and dura mater, epidural space was the simplest to be recognized. Among the other four tissues, interspinous ligament showed the most obvious imaging features, including the maximum penetration depth and the clear transverse stripes due to the thick fiber structure. Compared to other tissue types, ligamentum flavum showed higher imaging brightness close to the surface and the shallowest imaging depth. Imaging depths of fat and spinal cord were similar, but the imaging intensity of fat was not as evenly distributed as spinal cord. The corresponding histology results were also included in Fig. 1B. These tissues presented different cellular structures and distributions and correlated well with their OCT results except for fat. The fat tissue was featured with pockets of adipocytes in the histology, while this feature was not clear in the OCT results. This may be caused by the tissue compression we applied to mimic the clinical insertion scenario.

### Multi-class classification of OCT images by tissue layers using sequential binary method

OCT images of the five tissue layers were classified using CNN models based on three architectures, including: ResNet50^52^, Xception^54^ and Inception^55^. The prediction accuracies of the three models were shown in Supplementary Table 1. Its corresponding average multi-class confusion matrix was shown in Supplementary Table 2. The detailed sevenfold cross-validation results using ResNet50, Xception and Inception were shown in Supplementary Tables 3–5, respectively. The corresponding average cross-validation ROC curves of Inception was shown in Supplementary Figure 1. However, the overall accuracies of the multi-class classification models based on Inception reached ~ 66%. Although this was significantly higher than the accuracy of 20% by random guessing, further improvement was needed for clinical use.

Since the multi-class classification results were not satisfactory, herein we proposed to use sequential binary methods to improve the classification accuracies. During the needle placement, the needle was inserted through fat, interspinous ligament, and ligamentum flavum until reaching the epidural space. Continuing the needle insertion beyond the epidural space can puncture the dura and damage the spinal cord. The classification process was thus divided into a sequential process of four binary classifications: (1) fat vs interspinous ligament; (2) interspinous ligament vs ligamentum flavum; (3) ligamentum flavum vs epidural space; and (4) epidural space vs spinal cord. A flowchart of the sequential binary classifications was shown in the Supplementary Figure 2. The prediction results were shown in the Table 1.

**Table 1:** Average accuracies and standard error based on the practical tissue layer sequence during puncture for cross-validation.

Testing fold	Fat vs Ligament			Ligament vs Flavum		
	ResNet50	Xception	Inception	ResNet50	Xception	Inception
S1	95.1% ± 2.2%	82.8% ± 8.5%	90.4% ± 2.2%	97.9% ± 1.7%	98.5% ± 1.2%	97.8% ± 2.0%
S2	93.6% ± 2.4%	88.2% ± 6.5%	92.4% ± 2.1%	98.7% ± 1.2%	98.9% ± 1.0%	98.9% ± 1.0%
S3	94.5% ± 2.5%	84.4% ± 6.5%	88.0% ± 2.4%	99.4% ± 0.5%	99.6% ± 0.3%	99.0% ± 0.5%
S4	90.9% ± 2.9%	84.7% ± 6.3%	89.7% ± 2.8%	98.5% ± 1.3%	98.7% ± 0.8%	97.7% ± 1.9%
S5	89.9% ± 2.3%	86.9% ± 4.7%	89.3% ± 2.5%	98.4% ± 1.4%	98.4% ± 0.9%	98.0% ± 1.5%
S6	90.6% ± 3.7%	82.3% ± 8.5%	89.9% ± 2.2%	98.8% ± 0.9%	98.2% ± 1.2%	97.8% ± 1.9%
S7	90.7% ± 3.5%	86.0% ± 3.3%	88.1% ± 3.0%	98.4% ± 1.4%	99.1% ± 0.7%	97.0% ± 2.7%
S8	88.7% ± 3.8%	82.7% ± 6.3%	86.3% ± 2.7%	98.7% ± 1.1%	98.8% ± 0.6%	98.7% ± 0.8%
Average	91.8% ± 1.0%	84.7% ± 2.2%	89.3% ± 2.2%	98.6% ± 0.4%	98.8% ± 0.3%	98.1% ± 0.6%

Testing fold	Flavum vs Epidural Space			Epidural Space vs Spinal Cord		
	ResNet50	Xception	Inception	ResNet50	Xception	Inception
S1	100.0% ± 0.0%	100.0% ± 0.0%	100.0% ± 0.0%	100.0% ± 0.0%	100.0% ± 0.0%	100.0% ± 0.0%
S2	100.0% ± 0.0%	100.0% ± 0.0%	100.0% ± 0.0%	100.0% ± 0.0%	100.0% ± 0.0%	100.0% ± 0.0%
S3	100.0% ± 0.0%	100.0% ± 0.0%	100.0% ± 0.0%	100.0% ± 0.0%	100.0% ± 0.0%	100.0% ± 0.0%
S4	100.0% ± 0.0%	100.0% ± 0.0%	100.0% ± 0.0%	100.0% ± 0.0%	100.0% ± 0.0%	100.0% ± 0.0%
S5	100.0% ± 0.0%	100.0% ± 0.0%	100.0% ± 0.0%	100.0% ± 0.0%	100.0% ± 0.0%	100.0% ± 0.0%
S6	100.0% ± 0.0%	100.0% ± 0.0%	100.0% ± 0.0%	100.0% ± 0.0%	100.0% ± 0.0%	100.0% ± 0.0%
S7	100.0% ± 0.0%	100.0% ± 0.0%	100.0% ± 0.0%	100.0% ± 0.0%	100.0% ± 0.0%	100.0% ± 0.0%
S8	100.0% ± 0.0%	100.0% ± 0.0%	100.0% ± 0.0%	100.0% ± 0.0%	100.0% ± 0.0%	100.0% ± 0.0%
Average	100.0% ± 0.0%	100.0% ± 0.0%	100.0% ± 0.0%	100.0% ± 0.0%	100.0% ± 0.0%	100.0% ± 0.0%

Overall, ResNet50 showed the best prediction results. The average cross-validation performance of the four binary classifications was shown in Supplementary Table 6. Table 2 further showed the test accuracy of the best-performing model (ResNet50) in each of the 8 testing folds and almost all the results were over 90%. There was substantial variability in the test accuracy among different subjects especially for the prediction accuracy of “Fat vs Interspinous Ligament”. While three subjects had test accuracies higher than 98.8%, the subjects in the S2 fold had the lowest test accuracy of 67.3%. This may be due to the tissue variability among different back bone samples and the different tissue compression during imaging especially considering fat is subject to tissue compression. Additionally, the representative ROC curves were shown in Supplementary Figure 3. The areas under the ROC curve (AUC) differed among different samples. The detailed confusion matrices for cross testing using ResNet50 average and standard error were shown in Supplementary Table 7–10.

**Table 2:** Average and standard error for cross-testing for the four binary comparisons for ResNet50.

Testing fold	Fat vs Ligament	Ligament vs Flavum	Flavum vs Epidural Space	Epidural Space vs Spinal Cord	Average
S1	81.3%	98.8%	100.0%	100.0%	95.0% ± 4.6%
S2	67.3%	98.0%	100.0%	100.0%	91.3% ± 8.0%
S3	92.0%	87.7%	98.8%	100.0%	94.6% ± 2.9%
S4	99.9%	99.4%	100.0%	100.0%	99.8% ± 0.1%
S5	98.8%	99.4%	100.0%	100.0%	99.6% ± 0.3%
S6	79.5%	99.0%	100.0%	100.0%	94.6% ± 5.1%
S7	94.2%	99.8%	100.0%	100.0%	98.5% ± 1.4%
S8	98.8%	100.0%	100.0%	100.0%	99.7% ± 0.3%
Average	89.0% ± 4.2%	97.8% ± 1.5%	99.8% ± 0.2%	100.0% ± 0.0%	96.65% ± 1.32%

**Figure 2:**
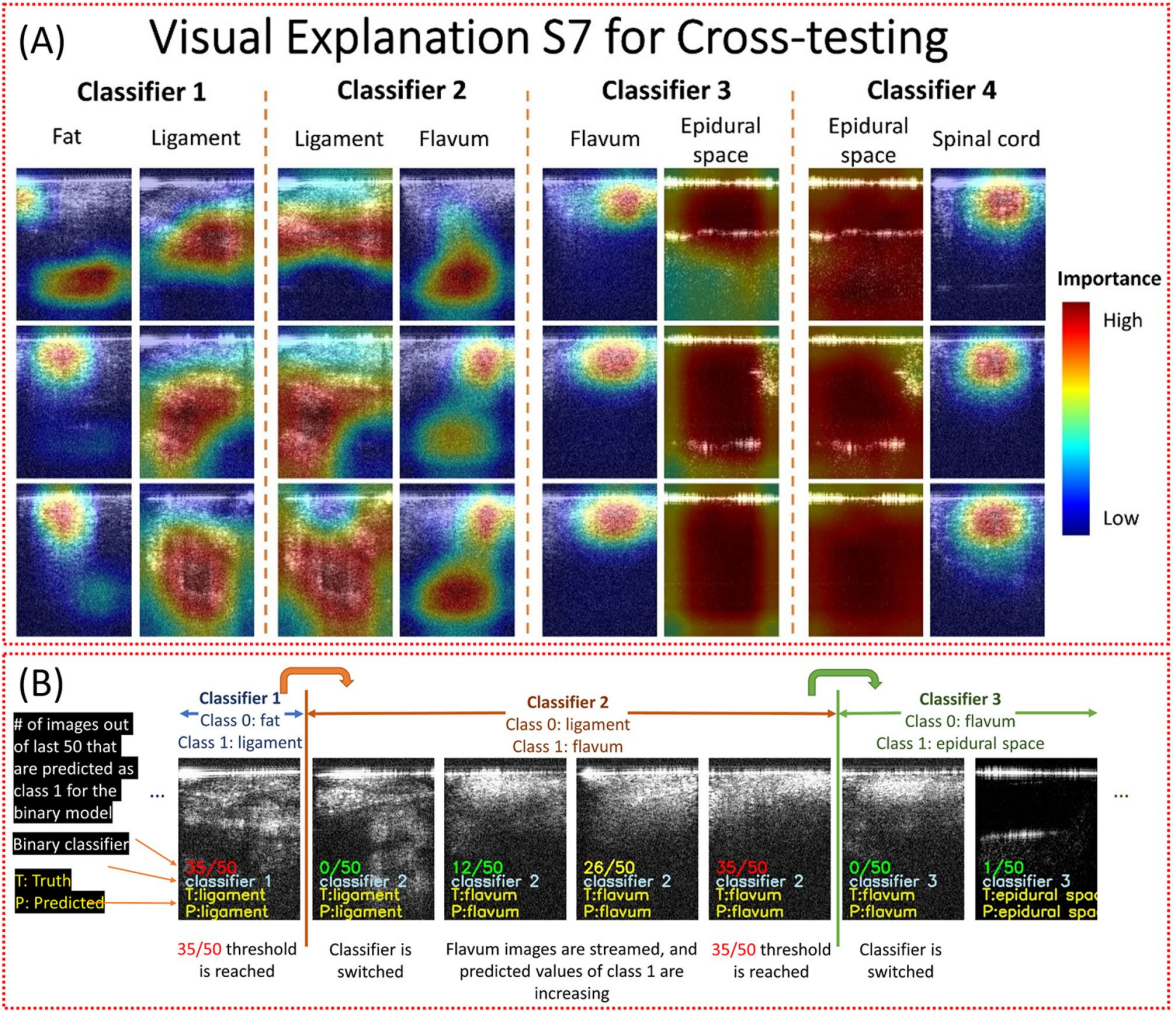
Class activation heatmaps for Subject 7 using ResNet50 in cross-testing (**A**) and video captures of the insertion process (**B**).

Class activation heatmaps of ResNet50 models were created for representative images to show the salient features used for classification (Fig. 2A). Each binary classification model paid attention to different regions of the images. For example, the black empty space was important for the models to recognize the epidural space images. A video stream of the OCT images was used to demonstrate the sequential binary models. The number of images was 100, 700, 100, 100, and 150 for fat, interspinous ligament, ligamentum flavum, epidural space and spinal cord, respectively, which was proportional to the width of these tissue layers^56–60^. After the binary classifier of fat vs. ligament detects 35 interspinous ligament images in the last 50 images, the needle was considered to be in the interspinous ligament and the next binary classifier of interspinous ligament vs. ligamentum flavum was activated to detect the upcoming ligamentum flavum. This simple logic was used to switch all the subsequent classifiers. Figure 2B showed some images from a video that can be found in the Github repository. Scenes from the video showing the switch from classifier 2 to classifier 3 and its arrival to epidural space. Each image showed three important pieces of information. First, the proportion of the last 50 images that were predicted to belong to Class 1, e.g., Class 1 was interspinous ligament in the first Classifier and was ligamentum flavum in the second Classifier. Initially, when the number of images was less than 50, the denominator shows the total number of images. Additionally, the color of fraction followed traffic lights colors. It changed from green to yellow at 26 and from yellow to red at 35. The second information was the current classifier. The last information was the truth and predicted label. The switch of binary classifier occurred when the number of images predicted as Class 1 reached 35. The fraction did not appear anymore when the last classifier was reached.

### Estimation of the distance between the needle tip and dura mater by regression

Inception, ResNet50, and Xception were compared for the regression task of estimating the distance of the needle tip to the dura mater. In Table 3, the mean and standard error of the cross-validation mean absolute percentage error (MAPE) for ResNet50, Xception, and Inception in all testing folds were shown. In every fold, the Inception model outperformed the ResNet50 and Xception models, indicated by the lowest MAPE.

**Table 3:** The average loss for each model type in cross-validation for each testing fold.

Testing folds	ResNet50	Xception	Inception
S1	3.71% ± 0.91%	3.99% ± 1.14%	3.13% ± 0.60%
S2	4.04% ± 0.99%	3.84% ± 0.98%	3.42% ± 0.82%
S3	3.82% ± 0.88%	3.62% ± 0.78%	3.09% ± 0.69%
S4	3.97% ± 1.34%	4.67% ± 1.68%	3.31% ± 0.82%
S5	3.88% ± 0.84%	4.15% ± 1.08%	3.23% ± 0.60%
S6	3.89% ± 0.82%	4.71% ± 1.31%	3.41% ± 0.83%
S7	3.88% ± 1.31%	4.51% ± 1.61%	3.81% ± 1.25%
S8	2.77% ± 0.32%	3.33% ± 0.39%	2.61% ± 0.37%
Average	3.74% ± 0.14%	4.10% ± 0.18%	3.25% ± 0.12%

**Figure 3:**
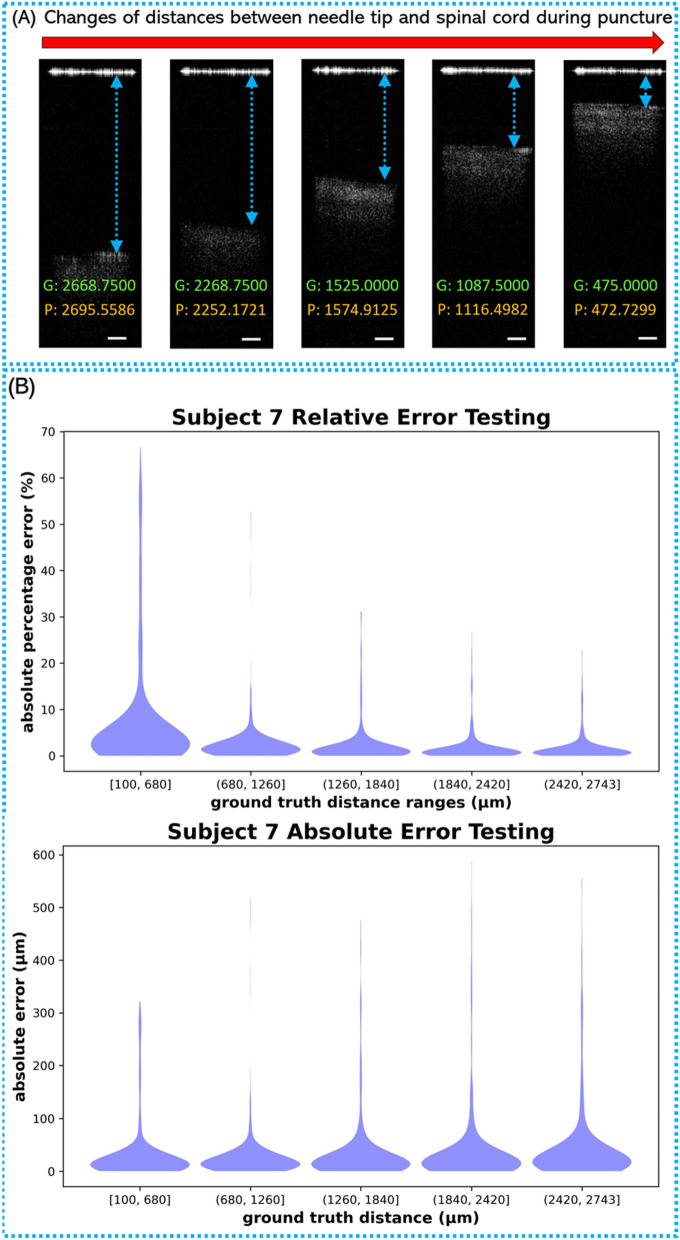
(**A**) Examples of epidural space images with different distances between needle tip and spinal cord surface. G: labeled ground truth value (μm); P: prediction value (μm); Scale bar: 250 μm. (**B**) The distribution of the predicted absolute percentage errors and absolute error in testing fold 7 with 3000 testing images.

In each testing rotation, a new Inception model was trained using all the images in the seven cross-validation folds and then evaluated on the unseen testing images in the one testing fold. Examples of OCT images with different distances between needle tip and tissue were shown in Fig. 3A. A model was trained on 21,000 images belonging to subjects 1, 2, 3, 4, 5, 6, and 8, and tested on 3,000 images belonging to subject 7. The distribution of the errors from the Inception model during the seventh testing fold (i.e., testing images belong to subject 7) can be visualized with the violin plots in Fig. 3B. The MAPE on this testing set was 3.626%, and the mean absolute error (MAE) was 34.093 μm. From the testing results on the Inception architecture, it was evident that the regression model can accurately estimate the distance to the dura mater in most of the OCT images. The distribution of the errors from the Inception model from all the other testing folds can be found in Supplementary Figure 5–6.

## Discussion

In this study, we validated our endoscopic OCT system for epidural anesthesia surgery guidance. The OCT endoscope can provide 10–100 times higher resolution than conventional medical imaging modalities. Moreover, this proposed endoscopic OCT system is compatible with the clinical-used epidural guiding methods (*e.g.*, ultrasound, fluoroscopy, and CT), and will complement these macroscopic methods by providing the detailed images in front of the epidural needle.

Five different tissue layers including fat, interspinous ligament, ligamentum flavum, epidural space and spinal cord were imaged. To assist the OCT image interpretation, a deep learning-based CAD platform was developed to automatically differentiate the tissue layers at the epidural needle tip and predict the distance from the needle tip to dura mater.

Three convolutional neural network architectures, including ResNet50, Xception and Inception, were tested for image classification and distance regression. The best classification accuracy of the five tissue layers were 60–65% from a multi-class Inception classifier. The main challenge was the differentiation between fat and spinal cord (Supplementary Table 2) because they had similar feature in OCT images (Fig. 1). Based on the needle puncture sequence, we divided the overall classification into four sequential binary classifications: Fat vs Interspinous Ligament; Interspinous Ligament vs Ligamentum Flavum; Ligamentum Flavum vs Epidural Space, and Epidural Space vs Spinal Cord. The overall prediction accuracies of all four classifications reached to more than 90%. ResNet50 presented the best overall performance compared to Xception and Inception. Due to the unique features of epidural space in OCT images, it was possible to achieve > 99% precision when the needle arrived the epidural space. Table 2 showed the accuracies of ~ 99.8% and 100% when classifying Epidural Space vs Ligamentum Flavum and Epidural Space vs Spinal Cord. This will allow accurate detection of the epidural space for injection of the anesthetic during epidural anesthesia. The sequential transition from one binary classifier to the next was controlled accurately using a simple logic, which was demonstrated in a video simulating the insertion of a needle through the five tissue layers (Fig. 2). In the future, this can be improved by combining CNN with Recurrent Neural Network (RNN) to handle the temporal dimension of video streaming data^61^. Additionally, we developed a CNN regression model to estimate the needle distance to the dura mater upon entry of the epidural space. For the regression task, Inception provided better performance compared to Xception and ResNet50. The mean relative error was 3.05%, which was able to track the accurate location of the needle tip in the epidural space.

CNNs have shown to be a valuable tool in biomedical imaging. Manually configuring CNN architectures for an imaging modality can be a tedious trial-and-error process. ResNet, Inception, and Xception are commonly used architectures for general image classification tasks. Here, we showed that the architectures can be easily adapted for both classification and regression tasks in biomedical imaging applications. The best performance was obtained by ResNet50 for the binary classifications and by Inception for the distance regression.

The nested-cross validation and testing procedure was computationally expensive, but it provided the uncertainty quantification of the test performance across subjects. The wall-clock time for training the binary classification models on NVIDIA Volta GPUs were ~ 11 min per validation fold for ResNet50, ~ 32 min per validation fold for Xception, and ~ 11 min per validation fold for Inception. The wall-clock time for training the regression models on NVIDIA RTX 3090 GPUs were ~ 50 min per validation fold for ResNet50, ~ 145 min per validation fold for Xception, and ~ 36 min per validation fold for Inception. The inferencing for the binary classifications on NVIDIA Volta GPUs took 13 ms per image on average. The inferencing for the distance regression on NVIDIA RTX 3090 GPUs took 2.1 ms per image on average. In future, the inferencing by these large CNN models can be further accelerated by weight pruning and knowledge distillation^62^.

In the next study, we will use the GRIN lens with a suitable diameter for practical 16-gauge Tuohy needle used in epidural anesthesia in our future hardware design^63,64^. Furthermore, we will miniaturize the size of our OCT scanner to make our system more portable and convenient for anesthesiologists to use in clinical applications. Finally, we will test the performance of our endoscopic OCT system together with the deep learning-based CAD platform in the in-vivo pig experiments. Difference of OCT images from in-vivo and ex-vivo condition may deteriorate the in-vivo testing results. In that case, we will re-train our model using in vivo pig data. Additionally, during the in-vivo experiments, there will be blood vessels surrounding the spinal cord^65^. To address this, we plan to further use Doppler OCT method for the blood vessel detection to avoid the rupture of blood vessels during epidural needle insertion.

## Method

### Experiment setup

**Figure 4:**
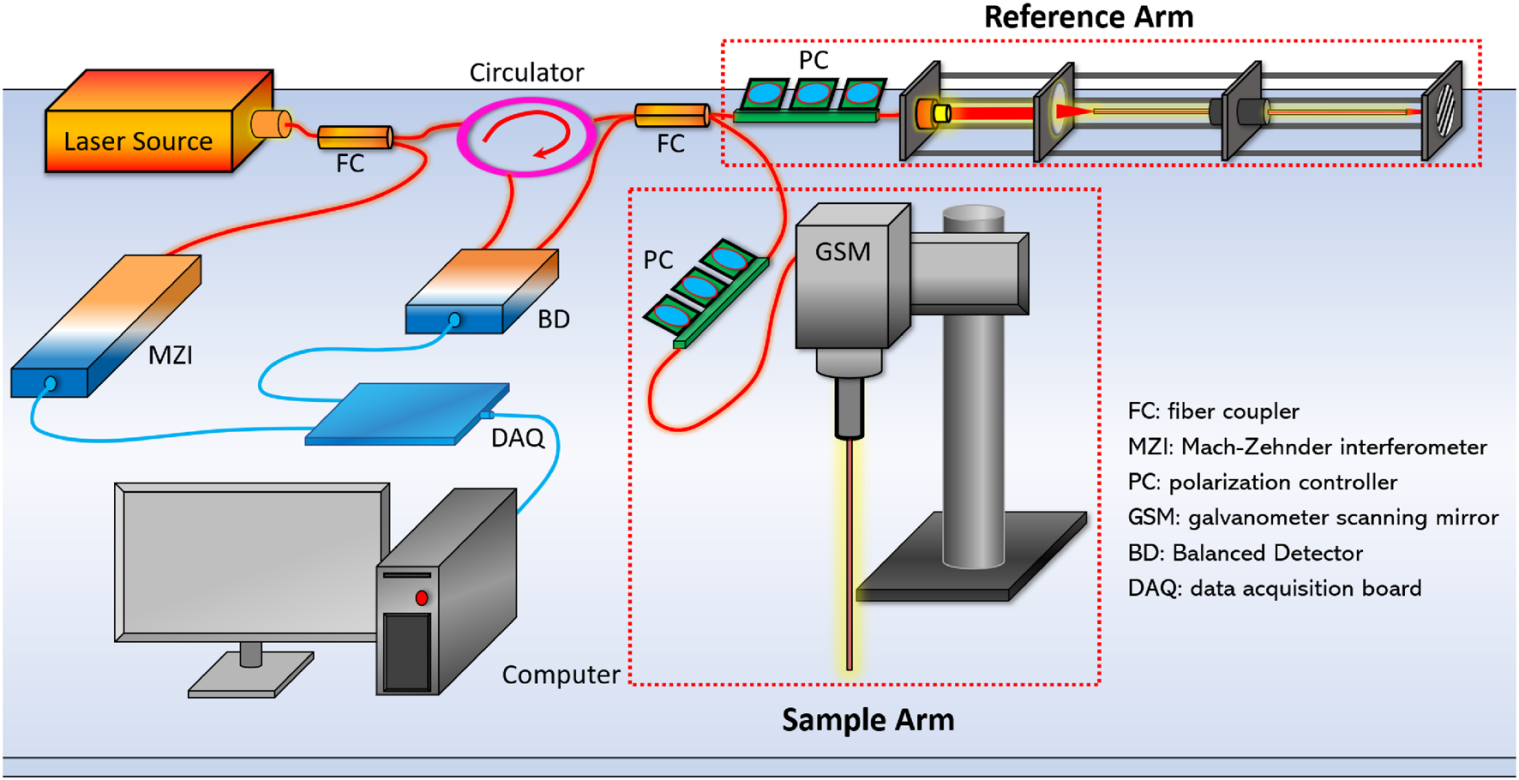
Schematic of forward-view OCT endoscope system.

The schematic of our forward-view OCT endoscope was shown in Fig. 4. Its working principle was based on a Michaelson interferometer with a reference arm and a sample arm^38^. The endoscopic system was built on a swept-source OCT (SS-OCT). The light source was a wavelength-swept laser with 1300 nm central wavelength and 100 nm bandwidth^66^. The laser had the maximum scanning speed at 200 kHz A-Scan rate. The light from the laser was first unevenly split by a fiber coupler (FC). 97% power was split into the circulator and transmitted into the interferometer, and the other 3% was input to the Mach–Zehnder interferometer (MZI) which provided the triggering signal for data sampling. The 97% power was further split by another 50:50 FC to the reference arm and the sample arm. The reflected signal from the reference arm and the backscattered signal from the sample arm interfered with each other and were collected by a balanced detector (BD) for noise reduction. The signal was then sent to data acquisition board (DAQ) and computer for post-processing based on Fourier transform^67^. While imaging the samples in the air, the axial resolution reached to 10.6 μm and the lateral resolution was 20 μm.

To achieve the endoscopic imaging, a gradient-index (GRIN) rod lens was added in the sample arm. It was fixed in front of the scanning lens of the galvanometer scanning mirror (GSM). The GRIN lens used in this study had a total length of 138 mm, an inner diameter of 1.3 mm, and a view angle of 11.0°. It was protected by a thin-wall steel tubing. For dispersion compensation, a second set of identical GRIN lens was stabilized in front of the reflector (mirror) of the reference arm. In addition, two polarization controllers (PC) were placed in each arm to reduce the noise level.

The GRIN lens utilized in the sample arm was assembled in front of the OCT scanning lens of the GSM. To decrease the reflection from the proximal end surface of the GRIN lens that significantly degraded the imaging quality, the proximal surface of the GRIN lens was aligned ~ 1.5 mm off the focus of the scanning lens. The GRIN lens had four integer pitch length to relay the images from the distal end to its proximal surface^68^. In the sample arm, the proximal GRIN lens surface was adjusted close to the focus point of the objective after the OCT scanner. Thus, the spatial information from the distal surface (tissue sample) of the GRIN lens transmitted to the proximal surface was further collected by the OCT scanner. Therefore, OCT images of the epidural tissues in front of the GRIN lens can be successfully obtained. Our endoscopic system provided ~ 1.25 mm field of view (FOV) with sensitivity of 92 dB.

### Data acquisition

**Figure 5:**
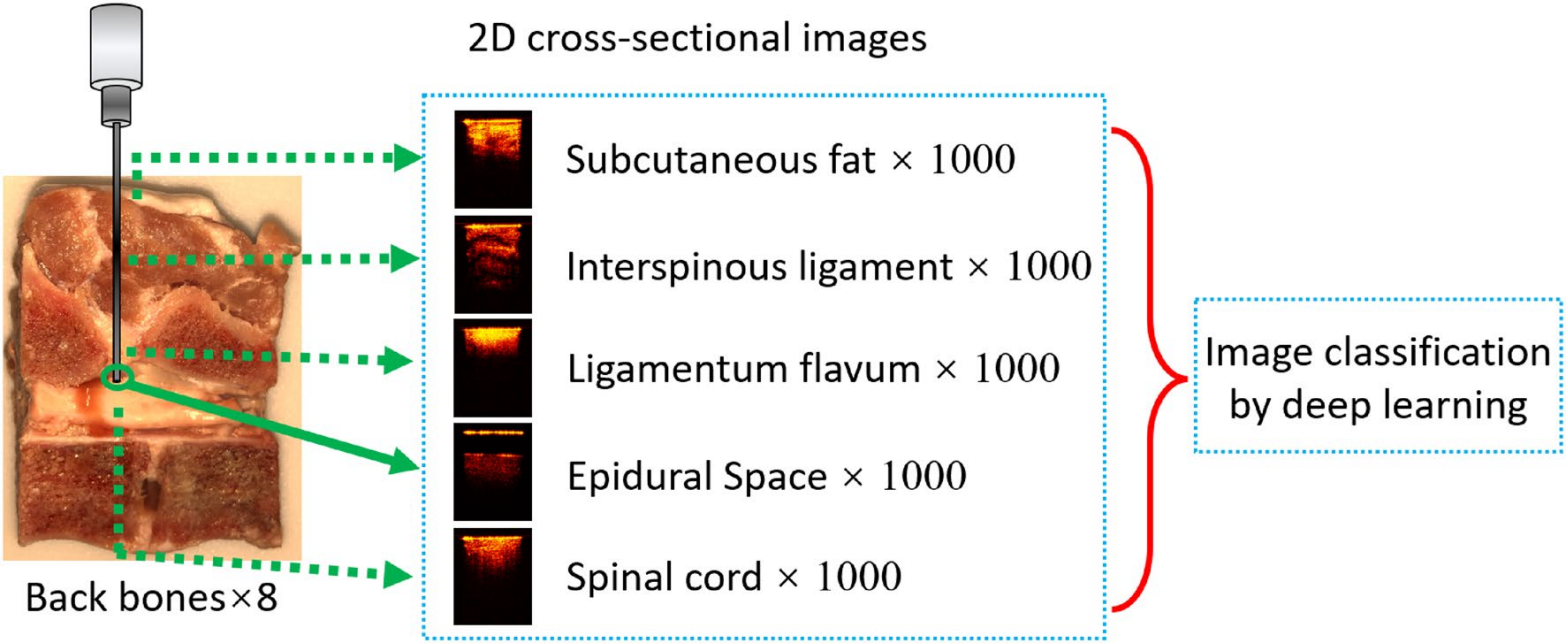
Data acquisition process.

Backbones from eight pigs were acquired from local slaughterhouses and cut at the middle before imaging to expose different tissue layers. From the cross-section of the sample, different tissue types could be clearly distinguished through the tissue anatomic features and their positions as shown in Fig. 5. To further limit the number of misclassified results, two lab members confirmed the tissue types before imaging started. In Fig. 5, five tissue layers including fat, interspinous ligament, ligamentum flavum, epidural space and spinal cord can be distinguished from their anatomic appearance. The OCT needle was placed against these confirmed tissue layers to obtain their OCT structural images. Following the practice of epidural needle placement, we mimicked the puncturing process by inserting the OCT endoscope through fat, interspinous ligament, ligamentum flavum and epidural space of our sample. Since the targeted position of the anesthetic injection is the epidural space with width ~ 1–6 mm^69^, we also obtained OCT images of epidural space by positioning the needle tip in front of the spinal cord at different distances. To mimic the condition of accidental puncture into spinal cord, we took OCT images while inserting the endoscope into the spinal cord. Some force was applied during imaging the four tissue types (fat, interspinous ligament, ligamentum flavum, and spinal cord) to generate compression to better represent the actual in-vivo clinical situation.

For each backbone sample, 1000 cross-sectional OCT images were obtained from each tissue layer. To decrease noise and increase the deep-learning processing speed, the original images were further cropped to smaller sizes that only contained the effective tissue information. Imaged were cropped to 181 × 241 pixels for the tissue classification. The data was uploaded to Zenodo (http://doi.org/10.5281/zenodo.5018581)^70^.

At the end of imaging, tissues of fat, interspinous ligament, ligamentum flavum and spinal cord with dura mater of the porcine back bones were excised and processed for histology following the same orientation of OCT endoscope imaging to compare with corresponding OCT results. The tissues were fixed with 10% formalin, embedded in paraffin, sectioned (4 µm thick) and stained with hematoxylin and eosin (H & E) for histological analysis. Images were analyzed by Keyence Microscope BZ-X800. Sectioning and H & E staining was carried out by the Tissue Pathology Shared Resource, Stephenson Cancer Center (SCC), University of Oklahoma Health Sciences Center. The Hematoxylin (cat# 3801571) and Eosin (cat# 3801616) were purchased from Leica biosystems, and the staining was performed utilizing Leica ST5020 Automated Multistainer following the HE staining protocol at the SCC Tissue Pathology core.

### Convolutional neural networks

Convolutional Neural Networks (CNN) were used to classify OCT images by epidural layers. Three CNN architectures, including ResNet50^52^, Inception^51^ and Xception^53^, were imported from the Keras library^71^. The output layer of the models was a dense layer that represented the number of categories. The images were centered by subtracting training mean pixel value. The SGD with Nesterov momentum optimizer was used with a learning rate of 0.01, a momentum of 0.9, and a decay of 0.01. The batch size was 32. Early stopping was used with a patience of 10. The loss function used was sparse categorical cross entropy.

Nested cross-validation and testing^72,73^ were used for model selection and benchmarking as described previously^66^. This evaluation strategy provided an unbiased estimation of model performance with uncertainty quantification using two nested loops for cross-validation and cross-testing. Images were acquired from eight subjects in this dataset. The images were divided to 8 folds by subjects to account for the subject-to-subject variability. An eight-fold cross-testing loop was performed by rotating through every subject for testing and using the remaining seven subjects (7000 images) for cross-validation. In the cross-validation, six subjects were used for training and one subject for validation in each rotation. The sevenfold cross-validation loop was used to compare the performance of three architecture models: ResNet50, Xception and Inception. The model with the best cross-validation performance was automatically selected for performance benchmarking in the corresponding testing fold. Supplementary Figure 7 depicted this evaluation strategy with Subject 1 used for testing. The performance of this overall procedure was evaluated by aggregating the testing performance from all 8 testing folds. Grad-CAM^74^ was used to generate instance-wise explanation of selected models^75,76^.

The computation was performed using the Schooner supercomputer at the University of Oklahoma and the Summit supercomputer at Oak Ridge National Laboratory. The computation on Schooner used five computational nodes, each of which had 40 CPU cores (Intel Xeon Cascade Lake) and 200 GB of RAM. The computation on Summited used up to 10 nodes, each of which had 2 IBM POWER9 processors and 6 NVIDIA Volta Graphic cards. The complete code for the classification models can be found at https://github.com/thepanlab/Endoscopic_OCT_Epidural.

The classification accuracy of the models was computed as:

1 $$Accuracy = \frac{TP +TN}{TP+TN+FP+FN}$$

where TP was True Positives, TN was True Negatives, FP was False Positives, and FN was False Negatives.

Receiver Operating Characteristic (ROC) curves were used to visualize the relationship between sensitivity and specificity. The area under the curve (AUC) of ROC was also used to assess the overall performance of the models.

### Epidural distance prediction using deep learning

OCT images of epidural space were obtained at a range of distances between approximately 0.2 mm and 2.5 mm from the needle tip to the spinal cord surface (dura mater). A total of 24,000 images from eight subjects were used for this task. For each image taken in the epidural space for the distance estimation task, the distance in micrometers (μm) from the epidural needle to the dura mater was manually calculated and labeled. This distance label served as the ground truth for computing the loss during the training process in the regression model. All images were of 241 × 681 pixels on X and Z (depth) axes with pixel size of 6.25 µm. The pixel values for each image were scaled in the range of 0–255.

The regression model was developed to estimate the distance from the epidural needle to the dura upon entry into the epidural space automatically. Three architectures, including ResNet50, Inception, and Xception, were compared using nested cross-validation and testing as described above. The final output layer consisted of a single neuron with an identity activation function for regression on the continuous distance values^77^. The SGD algorithm with Nesterov momentum optimization was used with a learning rate of 0.01, momentum of 0.9, and a decay rate of 0.01. Training took place with a batch size of 32 over 20 epochs. The mean absolute percentage error (MAPE) and mean absolute error (MAE) were the metrics used to evaluate the regression performance due to their intuitive interpretability in relation to the relative error. The MAPE and MAE performance metrics are defined in Eqs. (2) and (3), respectively. Model training and testing for the regression task was performed on a workstation equipped with dual NVIDIA RTX 3090 GPUs. The complete code for the regression models can be found at: https://github.com/thepanlab/Endoscopic_OCT_Epidural.

The classification accuracy of the models was computed as:

2 $$MAPE(\mathrm{\%})=\frac{100\mathrm{\%}}{n}\sum_{i=1}^{n}\frac{\left|{Y}_{i}-{X}_{i}\right|}{{Y}_{i}}$$

3 $$MAE=\frac{1}{n}\sum_{i=1}^{n}|{Y}_{i}-{X}_{i}|$$

where ${Y}_{i}$ was the ground truth distance, ${X}_{i}$ was the predicted distance, and $n$ was the number of OCT images.

## Supplementary Information

Supplementary Information 1.

Supplementary Video 1.

## Data Availability

The datasets generated and/or analyzed during the current study are available in the Github repository, https://github.com/thepanlab/Endoscopic_OCT_Epidural.

## References

[1] Moraca RJ, Sheldon DG, Thirlby RC (2003). The role of epidural anesthesia and analgesia in surgical practice. Ann. Surg..

[2] Svircevic V (2011). Meta-analysis of thoracic epidural anesthesia versus general anesthesia for cardiac surgery. Anesthesiology.

[3] Hollmann MW, Wieczorek KS, Smart M, Durieux ME (2001). Epidural anesthesia prevents hypercoagulation in patients undergoing major orthopedic surgery. Reg. Anesth. Pain Med..

[4] Hadimioglu N (2012). Combination of epidural anesthesia and general anesthesia attenuates stress response to renal transplantation surgery. Transplant Proc..

[5] Carli F, Trudel JL, Belliveau P (2001). The effect of intraoperative thoracic epidural anesthesia and postoperative analgesia on bowel function after colorectal surgery—A prospective, randomized trial. Dis. Colon Rectum.

[6] Li Y, Dong H, Tan S, Qian Y, Jin W (2019). Effects of thoracic epidural anesthesia/analgesia on the stress response, pain relief, hospital stay, and treatment costs of patients with esophageal carcinoma undergoing thoracic surgery: A single-center, randomized controlled trial. Medicine (Baltimore).

[7] Pesteie M, Lessoway V, Abolmaesumi P, Rohling RN (2018). Automatic localization of the needle target for ultrasound-guided epidural injections. IEEE Trans. Med. Imaging.

[8] Scott DB (1997). Identification of the epidural space: Loss of resistance to air or saline?. Reg. Anesth..

[9] Turnbull DK, Shepherd DB (2003). Post-dural puncture headache: Pathogenesis, prevention and treatment. Br. J. Anaesth..

[10] Horlocker TT (2000). Complications of spinal and epidural anesthesia. Anesthesiol. Clin. North Am..

[11] Horlocker TT, Wedel DJ (2000). Neurologic complications of spinal and epidural anesthesia. Reg. Anesth. Pain Med..

[12] Apfel CC (2010). Prevention of postdural puncture headache after accidental dural puncture: a quantitative systematic review. Br. J. Anaesth..

[13] Wu CL (2006). Gender and post-dural puncture headache. Anesthesiology.

[14] Hodges SD, Castleberg RL, Miller T, Ward R, Thornburg C (1998). Cervical epidural steroid injection with intrinsic spinal cord damage. Two case reports. Spine (Phila Pa 1976).

[15] Tripathi M, Nath SS, Gupta RK (2005). Paraplegia after intracord injection during attempted epidural steroid injection in an awake-patient. Anesth. Analg..

[16] Horlocker TT, McGregor DG, Matsushige DK, Schroeder DR, Besse JA (1997). A retrospective review of 4767 consecutive spinal anesthetics: Central nervous system complications. Perioperative Outcomes Group. Anesth. Analg..

[17] Keohane M (1996). Patient comfort: spinal versus epidural anesthesia for cesarean section. Anesth. Analg..

[18] Rigg JRA (2002). Epidural anaesthesia and analgesia and outcome of major surgery: A randomised trial. Lancet.

[19] Hoffmann VLH (1999). Posterior epidural space depth: Safety of the loss of resistance and hanging drop techniques. Brit J Anaesth.

[20] Frumin MJ, Schwartz H, Burns JJ, Brodie BB, Papper EM (1953). Sites of sensory blockade during segmental spinal and segmental peridural anesthesia in man. Anesthesiology.

[21] Grondin LS (2009). Success of spinal and epidural labor analgesia comparison of loss of resistance technique using air versus saline in combined spinal-epidural labor analgesia technique. Anesthesiology.

[22] Honigmann, S. *et al.* In *Medical Imaging 2019: Image-Guided Procedures, Robotic Interventions, and Modeling.* 109510K (International Society for Optics and Photonics).

[23] Hermanides J, Hollmann MW, Stevens MF, Lirk P (2012). Failed epidural: causes and management. Br. J. Anaesth..

[24] McLeod A, Roche A, Fennelly M (2005). Case series: Ultrasonography may assist epidural insertion in scoliosis patients. Can. J. Anaesth..

[25] Stojanovic MP (2002). The role of fluoroscopy in cervical epidural steroid injections: an analysis of contrast dispersal patterns. Spine (Phila Pa 1976).

[26] Tang Q, Liang C-P, Wu K, Sandler A, Chen Y (2015). Real-time epidural anesthesia guidance using optical coherence tomography needle probe. Quant. Imaging Med. Surg..

[27] Saidman LJ, Eger EI (1965). Change in cerebrospinal fluid pressure during pneumoencephalography under nitrous oxide anesthesia. Anesthesiology.

[28] Cheng ACK (1994). Intended epidural-anesthesia as possible cause of cauda-equina syndrome. Anesth. Analg..

[29] Carter MI (1984). Cervical surgical emphysema following extradural analgesia. Anaesthesia.

[30] Mehta M, Sokoll MD, Gergis SD (1984). Effects of venous air embolism on the cardiovascular system and acid base balance in the presence and absence of nitrous oxide. Acta Anaesthesiol. Scand..

[31] Grau T, Leipold RW, Fatehi S, Martin E, Motsch J (2004). Real-time ultrasonic observation of combined spinal-epidural anaesthesia. Eur. J. Anaesth..

[32] Kang SY (2020). Advantages of the combination of conscious sedation epidural anesthesia under fluoroscopy guidance in lumbar spine surgery. J. Pain Res..

[33] Richardson J, Groen GJ (2005). Applied epidural anatomy. Contin. Educ. Anaesth. Crit. Care Pain.

[34] Ting CK, Chang Y (2010). Technique of fiber optics used to localize epidural space in piglets. Opt. Express.

[35] Lin SP (2012). Discriminant analysis for anaesthetic decision-making: An intelligent recognition system for epidural needle insertion. Br. J. Anaesth..

[36] Rathmell JP (2010). Identification of the epidural space with optical spectroscopy an in vivo swine study. Anesthesiology.

[37] Anderson TA, Kang JW, Gubin T, Dasari RR, So PT (2016). Raman spectroscopy differentiates each tissue from the skin to the spinal cord: A novel method for epidural needle placement?. Anesthesiology.

[38] Huang D (1991). Optical coherence tomography. Science.

[39] Li XD (2000). Optical coherence tomography: Advanced technology for the endoscopic imaging of Barrett's esophagus. Endoscopy.

[40] Carotenuto B (2017). Optical guidance systems for epidural space identification. Ieee J Sel Top Quant.

[41] Sharma GK (2015). Long-range optical coherence tomography of the neonatal upper airway for early diagnosis of intubation-related subglottic injury. Am. J. Respir. Crit. Care Med..

[42] Chen Y (2007). Ultrahigh resolution optical coherence tomography of Barrett's esophagus: Preliminary descriptive clinical study correlating images with histology. Endoscopy.

[43] Sergeev AM (1997). In vivo endoscopic OCT imaging of precancer and cancer states of human mucosa. Opt. Express.

[44] Kao MC, Wu YT, Tsou MY, Kuo WC, Ting CK (2018). Intelligent epidural needle placement using fiber-probe optical coherence tomography in a piglet model. Biomed. Opt. Express.

[45] Kuo WC (2015). Fiber-needle swept-source optical coherence tomography system for the identification of the epidural space in piglets. Anesthesiology.

[46] Litjens G (2017). A survey on deep learning in medical image analysis. Med. Image Anal..

[47] Shen D, Wu G, Suk H-I (2017). Deep learning in medical image analysis. Annu. Rev. Biomed. Eng..

[48] Wang C, Gan M, Zhang M, Li DY (2020). Adversarial convolutional network for esophageal tissue segmentation on OCT images. Biomed. Opt. Express.

[49] Rong Y (2019). Surrogate-assisted retinal OCT image classification based on convolutional neural networks. IEEE J. Biomed. Health Inform..

[50] Rasti R, Rabbani H, Mehridehnavi A, Hajizadeh F (2018). Macular OCT classification using a multi-scale convolutional neural network ensemble. IEEE Trans. Med. Imaging.

[51] Szegedy, C., Vanhoucke, V., Ioffe, S., Shlens, J. & Wojna, Z. In *Proceedings of the IEEE Conference on Computer Vision and Pattern Recognition. *2818–2826.

[52] He, K., Zhang, X., Ren, S. & Sun, J. In *Proceedings of the IEEE Conference on Computer Vision and Pattern Recognition.* 770–778.

[53] Chollet, F. In *Proceedings of the IEEE conference on computer vision and pattern recognition. *1251–1258.

[54] Kassani, S. H. *et al.* In *2019 IEEE International Symposium on Signal Processing and Information Technology (ISSPIT).* 1–6 (IEEE).

[55] Xia, X., Xu, C. & Nan, B. In *2017 2nd International Conference on Image, Vision and Computing (ICIVC).* 783–787 (IEEE).

[56] Rajput D, Srivastava AK, Kumar R (2010). Spinal epidural lipomatosis: An unusual cause of relapsing and remitting paraparesis. J. Pediatr. Neurosci..

[57] Frostell A, Hakim R, Thelin EP, Mattsson P, Svensson M (2016). A review of the segmental diameter of the healthy human spinal cord. Front Neurol.

[58] Waldman, S. D. in *Pain Management* (eds Waldman, S. D. & Bloch, J. I.) xiii (W.B. Saunders, 2007).

[59] Cramer GD, Darby SA, Cramer GD (2014). Clinical Anatomy of the Spine, Spinal Cord, and ANS.

[60] Jang D, Park S (2014). A morphometric study of the lumbar interspinous space in 100 stanford university medical center patients. J. Korean Neurosurg. Soc..

[61] Masood, S., Srivastava, A., Thuwal, H. C. & Ahmad, M. Real-Time Sign Language Gesture (Word) Recognition from Video Sequences Using CNN and RNN. In: *Intelligent Engineering Informatics*. Advances in Intelligent Systems and Computing, vol 695 (eds Bhateja, V. *et al.*) 623–632. 10.1007/978-981-10-7566-7_63 (Springer, Singapore, 2018).

[62] Aghli, N. & Ribeiro, E. In *Proceedings of the IEEE/CVF Conference on Computer Vision and Pattern Recognition.* 3191–3198.

[63] Joshi G, McCarroll S (1994). Evaluation of combined spinal-epidural anesthesia using two different techniques. Reg. Anesth..

[64] Magill JC (2010). A novel actuator for simulation of epidural anesthesia and other needle insertion procedures. Simul. Healthc..

[65] Hansen JT, Netter FH, Machado CAG (2019). Netter basic science 1 online resource (xviii, 555 pages).

[66] Wang C (2021). Deep-learning-aided forward optical coherence tomography endoscope for percutaneous nephrostomy guidance. Biomed. Opt. Express.

[67] Tang QG, Liang CP, Wu K, Sandler A, Chen Y (2015). Real-time epidural anesthesia guidance using optical coherence tomography needle probe. Quant. Imaging Med. Surg..

[68] Jung JC, Mehta AD, Aksay E, Stepnoski R, Schnitzer MJ (2004). In vivo mammalian brain imaging using one- and two-photon fluorescence microendoscopy. J. Neurophysiol..

[69] Manchikanti, L. & Atluri, S. in *Pain Management* (eds Steven D. Waldman & Joseph I. Bloch) 1281–1293 (W.B. Saunders, 2007).

[70] Wang, C., Tang, Q., Ton, N. B. T., Calle, P. & Pan, C. (Dataset on Zenodo, 2021).

[71] Keras (2015).

[72] Iizuka N (2003). Oligonucleotide microarray for prediction of early intrahepatic recurrence of hepatocellular carcinoma after curative resection. The Lancet.

[73] Krstajic D, Buturovic LJ, Leahy DE, Thomas S (2014). Cross-validation pitfalls when selecting and assessing regression and classification models. J. Cheminform..

[74] Selvaraju, R. R. *et al.* in *Proceedings of the IEEE international conference on computer vision.* 618–626.

[75] Chollet F (2018). Deep Learning with Python.

[76] Badre A, Pan C (2022). LINA: A linearizing neural network architecture for accurate first-order and second-order interpretations. IEEE Access.

[77] Xia P, Hu J, Peng Y (2018). EMG-based estimation of limb movement using deep learning with recurrent convolutional neural networks. Artif. Organs.

