# Supplementary material for: Epidural anesthesia needle guidance by forward-view endoscopic optical coherence tomography and deep learning
Source: Sci Rep. 2022 May 31;12:9057. doi: 10.1038/s41598-022-12950-7 (PMC9156706; doi:10.1038/s41598-022-12950-7)
Supplement: Supplementary file 1 — Supplementary Information 1. [file 41598_2022_12950_MOESM1_ESM.docx]

Epidural anesthesia needle guidance by forward-view endoscopic optical coherence tomography and deep learning: supplement document

Chen Wang,^a, #^ Paul Calle,^b, #^ Justin C. Reynolds,^b, #^ Sam Ton,^a^ Feng Yan,^a^ Anthony M. Donaldson,^a^ Avery D. Ladymon,^a^ Pamela R. Roberts,^c^ Alberto J. de Armendi,^c^ Kar-ming Fung,^d, e^ Shashank S. Shettar,^c^ Chongle Pan,^b^ Qinggong Tang,^a,f,*^

*aStephenson School of Biomedical Engineering, University of Oklahoma, Norman, OK 73019**, USA*

*bSchool of Computer Science, University of Oklahoma, Norman, OK 73019, USA*

*cDepartment of Anesthesiology, University of Oklahoma Health Sciences Center, Oklahoma City, OK 73104, USA*

*dDepartment of Pathology, University of Oklahoma Health Sciences Center, Oklahoma City, OK 73104, USA*

*eStephenson Cancer Center, University of Oklahoma Health Sciences Center, Oklahoma City, OK 73104, USA*

*fInstitute for Biomedical Engineering, Science, and Technology (IBEST), University of Oklahoma, Norman, OK 73019, USA*

*#These authors contributed equally to this work.*

Corresponding author: Qinggong Tang Ph.D.

173 Felgar St., Room 307.

Norman, OK, USA, 73019*.*

qtang@ou.edu

Table 1. The averages and standard errors of the validation accuracies

| **Testing folds** | **ResNet50** | **Xception** | **Inception** |
| --- | --- | --- | --- |
| **S1** | 67.02%±3.82% | 64.73%± 2.07% | 69.89%± 3.31% |
| **S2** | 67.19%±1.97% | 63.04%± 1.47% | 66.52%± 3.34% |
| **S3** | 64.86%±2.60% | 61.93%± 2.14% | 64.83%± 0.58% |
| **S4** | 67.51%±2.91% | 61.25%± 2.41% | 62.94%± 2.92% |
| **S5** | 66.10%±2.74% | 63.16%± 1.99% | 67.92%± 3.91% |
| **S6** | 61.09%±1.35% | 55.57%± 1.67% | 64.46%± 3.72% |
| **S7** | 62.49%±1.64% | 61.05%± 2.09% | 65.35%± 2.96% |
| **S8** | 65.63%±1.27% | 62.83%± 2.25% | 65.62%± 3.06% |
| **Average** | 65.24%±0.86% | 61.70%± 1.09% | 65.94%± 0.72% |

Table 2. Average multi-class confusion matrix with standard error for Inception for cross-validation.

|  | | **PREDICTED** | | | | |
| --- | --- | --- | --- | --- | --- | --- |
|  |  | **Fat** | **Interspinous Ligament** | **Flavum** | **Epidural space** | **Spinal Cord** |
| **TRUE** | **Fat** | **330** ± **34** | 108 ± 18 | 103 ± 17 | 0 ± 0 | 459 ± 39 |
|  | **Interspinous Ligament** | 116 ± 24 | **858 ± 24** | 2 ± 1 | 0 ± 0 | 24 ± 4 |
|  | **Ligamentum Flavum** | 161 ± 26 | 11 ± 4 | **675 ± 40** | 0 ± 0 | 153 ± 22 |
|  | **Epidural space** | 0 ± 0 | 0 ± 0 | 0 ± 0 | **1000 ± 0** | 0 ± 0 |
|  | **Spinal Cord** | 331 ± 28 | 50 ± 8 | 215 ± 24 | 0 ± 0 | **404 ± 30** |

Table 3. Training results of the 7-fold cross validation using 5-category ResNet50

| **Testing fold** | **S1_val** | **S2_val** | **S3_val** | **S4_val** | **S5_val** | **S6_val** | **S7_val** | **S8_val** | **Average** | **Std error** |
| --- | --- | --- | --- | --- | --- | --- | --- | --- | --- | --- |
| **S1** |  | 71.3% | 58.8% | 67.3% | 63.6% | 53.9% | 85.5% | 68.7% | **67.0%** | 3.8% |
| **S2** | 73.3% |  | 59.2% | 64.4% | 65.6% | 71.8% | 71.8% | 64.3% | **67.2%** | 2.0% |
| **S3** | 57.8% | 55.2% |  | 69.9% | 66.9% | 62.7% | 75.1% | 66.3% | **64.9%** | 2.6% |
| **S4** | 67.2% | 58.7% | 57.5% |  | 73.0% | 78.8% | 71.6% | 65.8% | **67.5%** | 2.9% |
| **S5** | 70.2% | 57.6% | 58.9% | 62.7% |  | 77.6% | 71.3% | 64.3% | **66.1%** | 2.7% |
| **S6** | 61.1% | 60.8% | 61.4% | 66.0% | 57.9% |  | 55.8% | 64.8% | **61.1%** | 1.3% |
| **S7** | 67.2% | 58.9% | 59.2% | 57.5% | 68.7% | 64.3% |  | 61.7% | **62.5%** | 1.6% |
| **S8** | 67.0% | 60.5% | 63.5% | 68.2% | 65.4% | 70.7% | 64.1% |  | **65.6%** | 1.3% |
| **Average** | **66.3%** | **60.4%** | **59.8%** | **65.2%** | **65.9%** | **68.5%** | **70.7%** | **65.1%** |  |  |

Table 4. Training results of the 7-fold cross validation using 5-category Xception

| **Testing fold** | **S1_val** | **S2_val** | **S3_val** | **S4_val** | **S5_val** | **S6_val** | **S7_val** | **S8_val** | **Average** | **Std error** |
| --- | --- | --- | --- | --- | --- | --- | --- | --- | --- | --- |
| **S1** |  | 66.6% | 56.2% | 71.7% | 52.5% | 64.4% | 78.4% | 63.2% | **64.7%** | 3.3% |
| **S2** | 65.7% |  | 44.6% | 71.3% | 59.9% | 66.6% | 67.9% | 65.3% | **63.0%** | 3.3% |
| **S3** | 62.7% | 61.1% |  | 60.7% | 60.1% | 61.5% | 62.9% | 64.5% | **61.9%** | 0.6% |
| **S4** | 63.4% | 50.9% | 54.3% |  | 74.8% | 64.7% | 60.5% | 60.1% | **61.3%** | 2.9% |
| **S5** | 68.0% | 44.2% | 53.8% | 66.0% |  | 73.3% | 70.0% | 66.8% | **63.2%** | 3.9% |
| **S6** | 61.7% | 54.5% | 35.6% | 64.4% | 58.6% |  | 52.0% | 62.1% | **55.6%** | 3.7% |
| **S7** | 67.1% | 52.5% | 49.6% | 72.1% | 61.4% | 63.4% |  | 61.2% | **61.1%** | 3.0% |
| **S8** | 58.6% | 54.9% | 55.7% | 61.3% | 63.1% | 78.0% | 68.2% |  | **62.8%** | 3.1% |
| **Average** | **63.9%** | **55.0%** | **50.0%** | **66.8%** | **61.5%** | **67.4%** | **65.7%** | **63.3%** |  |  |

Table 5. Training results of the 7-fold cross validation using 5-category Inception

| **Testing fold** | **S1_val** | **S2_val** | **S3_val** | **S4_val** | **S5_val** | **S6_val** | **S7_val** | **S8_val** | **Average** | **Std error** |
| --- | --- | --- | --- | --- | --- | --- | --- | --- | --- | --- |
| **S1** |  | 69.0% | 61.1% | 72.5% | 65.2% | 77.1% | 74.4% | 70.0% | **69.9%** | 2.1% |
| **S2** | 65.8% |  | 59.8% | 69.2% | 65.6% | 65.6% | 72.5% | 67.0% | **66.5%** | 1.5% |
| **S3** | 53.6% | 62.2% |  | 66.9% | 68.1% | 71.3% | 66.6% | 65.2% | **64.8%** | 2.1% |
| **S4** | 62.2% | 52.7% | 59.7% |  | 61.8% | 72.7% | 68.6% | 63.0% | **62.9%** | 2.4% |
| **S5** | 67.3% | 61.6% | 61.7% | 71.0% |  | 75.2% | 72.6% | 66.1% | **67.9%** | 2.0% |
| **S6** | 65.0% | 61.2% | 57.5% | 68.0% | 66.5% |  | 70.6% | 62.5% | **64.5%** | 1.7% |
| **S7** | 69.4% | 60.7% | 55.6% | 72.2% | 66.8% | 66.6% |  | 66.2% | **65.3%** | 2.1% |
| **S8** | 58.2% | 62.2% | 60.6% | 73.7% | 66.3% | 73.1% | 65.2% |  | **65.6%** | 2.3% |
| **Average** | **63.1%** | **61.4%** | **59.4%** | **70.5%** | **65.8%** | **71.6%** | **70.1%** | **65.7%** |  |  |


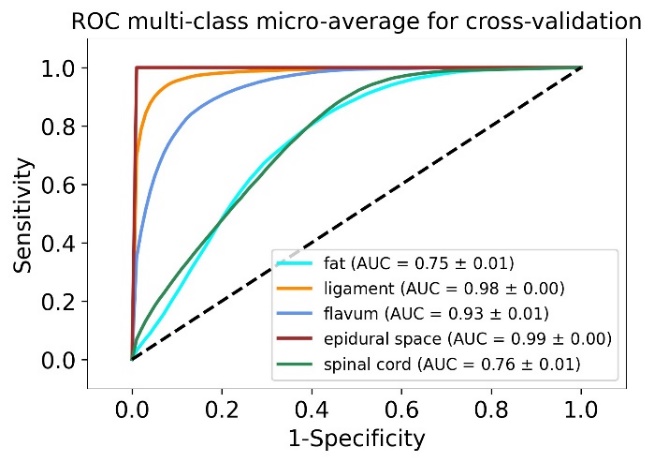


**Fig. 1.** Average ROC multi-class for validation in cross-validation Inception. Average and standard error for AUC are listed.


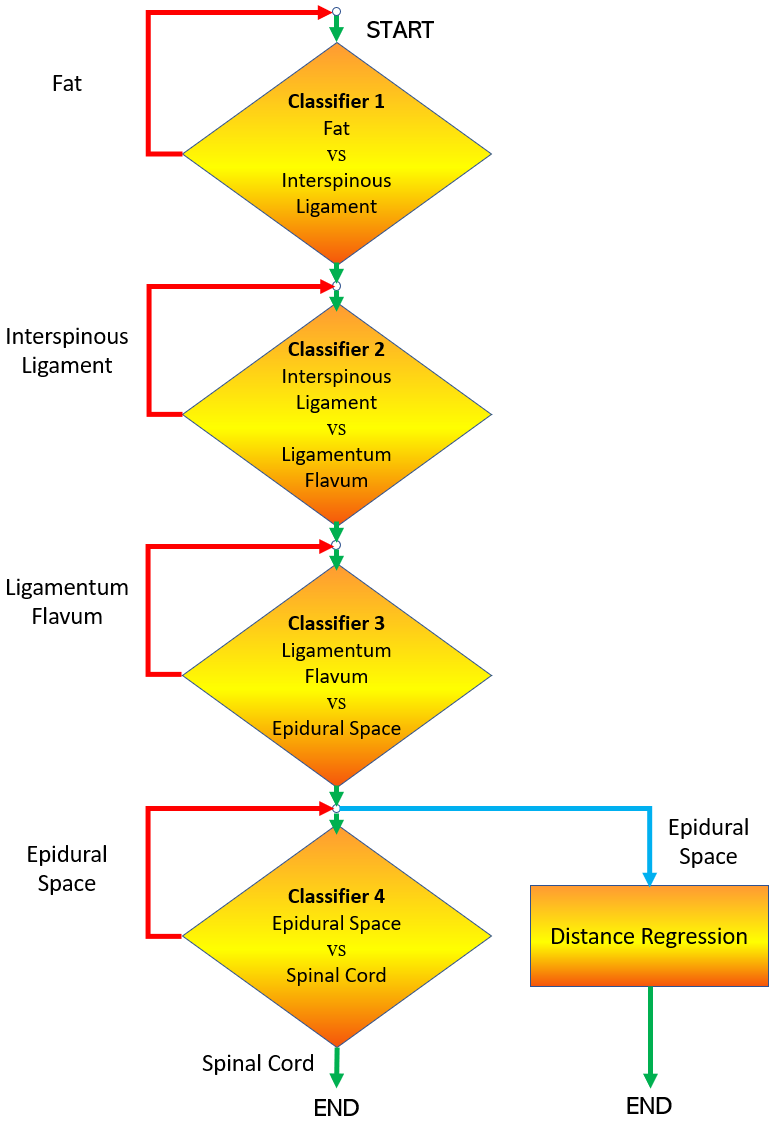


**Fig. 2.** Tissue layer classifications based on the puncturing sequence in epidural anesthesia

Table 6. Average and standard error for four binaries groups for cross-validation

| **Testing Fold** | **RN50** | **Xception** | **Inception** |
| --- | --- | --- | --- |
| **S1** | **98.24%±0.76%** | 95.31%± 2.50% | 97.06%± 1.03% |
| **S2** | **98.08%±0.82%** | 96.76%± 1.82% | 97.81%± 0.81% |
| **S3** | **98.47%±0.75%** | 95.98%± 2.00% | 96.76%± 1.14% |
| **S4** | **97.34%±1.05%** | 95.86%± 1.95% | 96.84%± 1.14% |
| **S5** | **97.09%±1.02%** | 96.31%± 1.54% | 96.84%± 1.09% |
| **S6** | **97.37%±1.18%** | 95.11%± 2.49% | 96.93%± 1.05% |
| **S7** | **97.30%±1.15%** | 96.30%± 1.38% | 96.27%± 1.35% |
| **S8** | **96.84%±1.30%** | 95.39%± 2.05% | 96.25%± 1.29% |


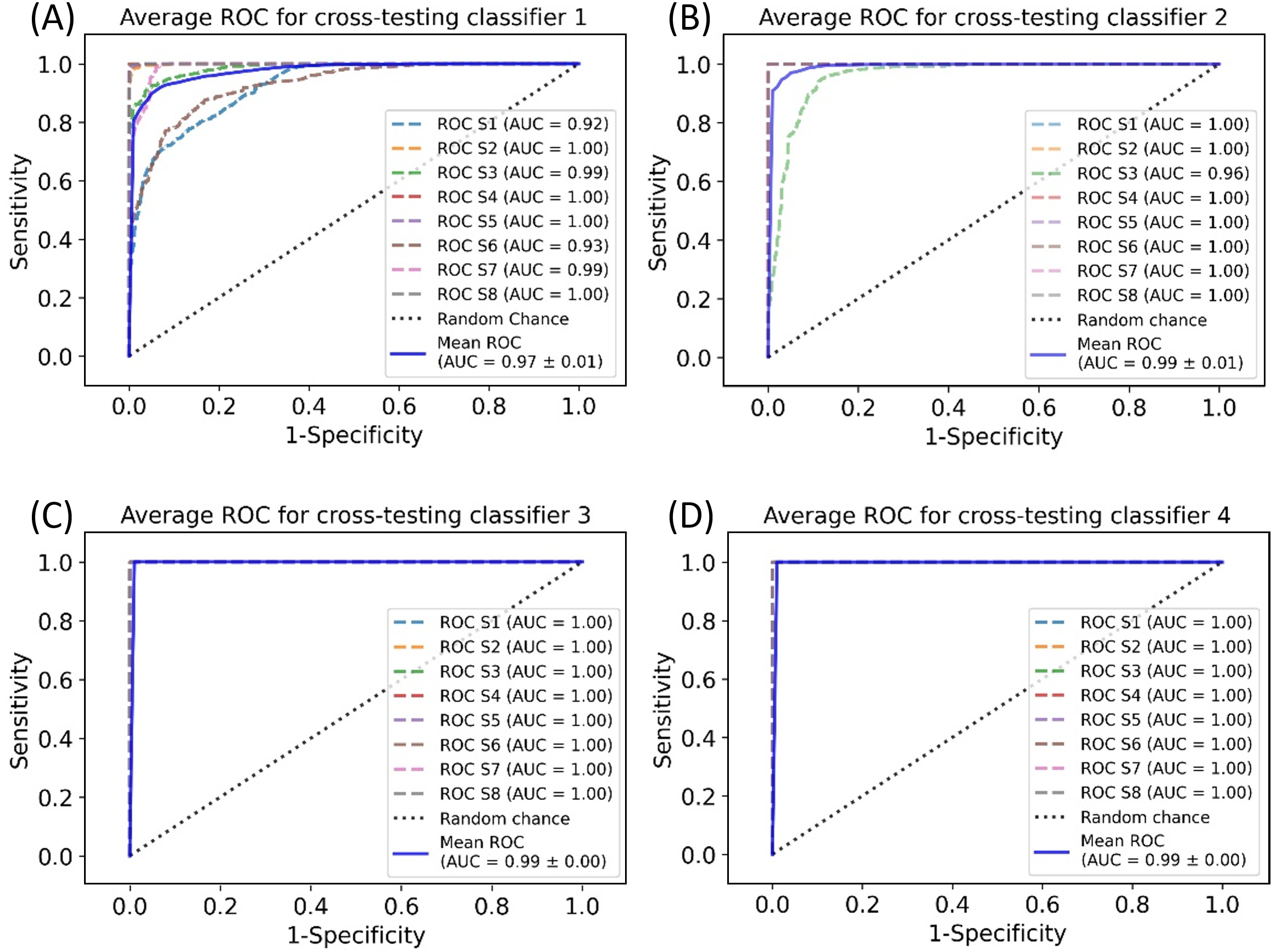


**Fig. 4.** Representative ROC curves in 1vs1 testing curves. (A) Classifier 1: fat vs interspinous ligament; (B) Classifier 2: interspinous ligament vs ligamentum flavum; (C) Classifier 3: ligamentum flavum vs epidural space; (D) Classifier 4: epidural space vs spinal cord.

Table 7. Confusion matrix for cross testing using ResNet50 classifier 1 (fat vs ligament)

|  |  | Predicted | |
| --- | --- | --- | --- |
|  |  | Fat | Ligament |
| Truth | Fat | 894±21 | 106±21 |
|  | Ligament | 115±30 | 885±30 |

Table 8. Confusion matrix for cross testing using ResNet50 classifier 2 (ligament vs flavum)

|  |  | Predicted | |
| --- | --- | --- | --- |
|  |  | Fat | Ligament |
| Truth | Fat | 982±3 | 18±3 |
|  | Ligament | 26±8 | 974±8 |

Table 9. Confusion matrix for cross testing using ResNet50 classifier 3 (flavum vs epidural space)

|  |  | Predicted | |
| --- | --- | --- | --- |
|  |  | Fat | Ligament |
| Truth | Fat | 997±1 | 3±1 |
|  | Ligament | 0±0 | 1000±0 |

Table 10. Confusion matrix for cross testing using ResNet50 classifier 4 (epidural space vs spinal cord) without subject 7

|  |  | Predicted | |
| --- | --- | --- | --- |
|  |  | Fat | Ligament |
| Truth | Fat | 1000±0 | 0±0 |
|  | Ligament | 0±0 | 1000±0 |


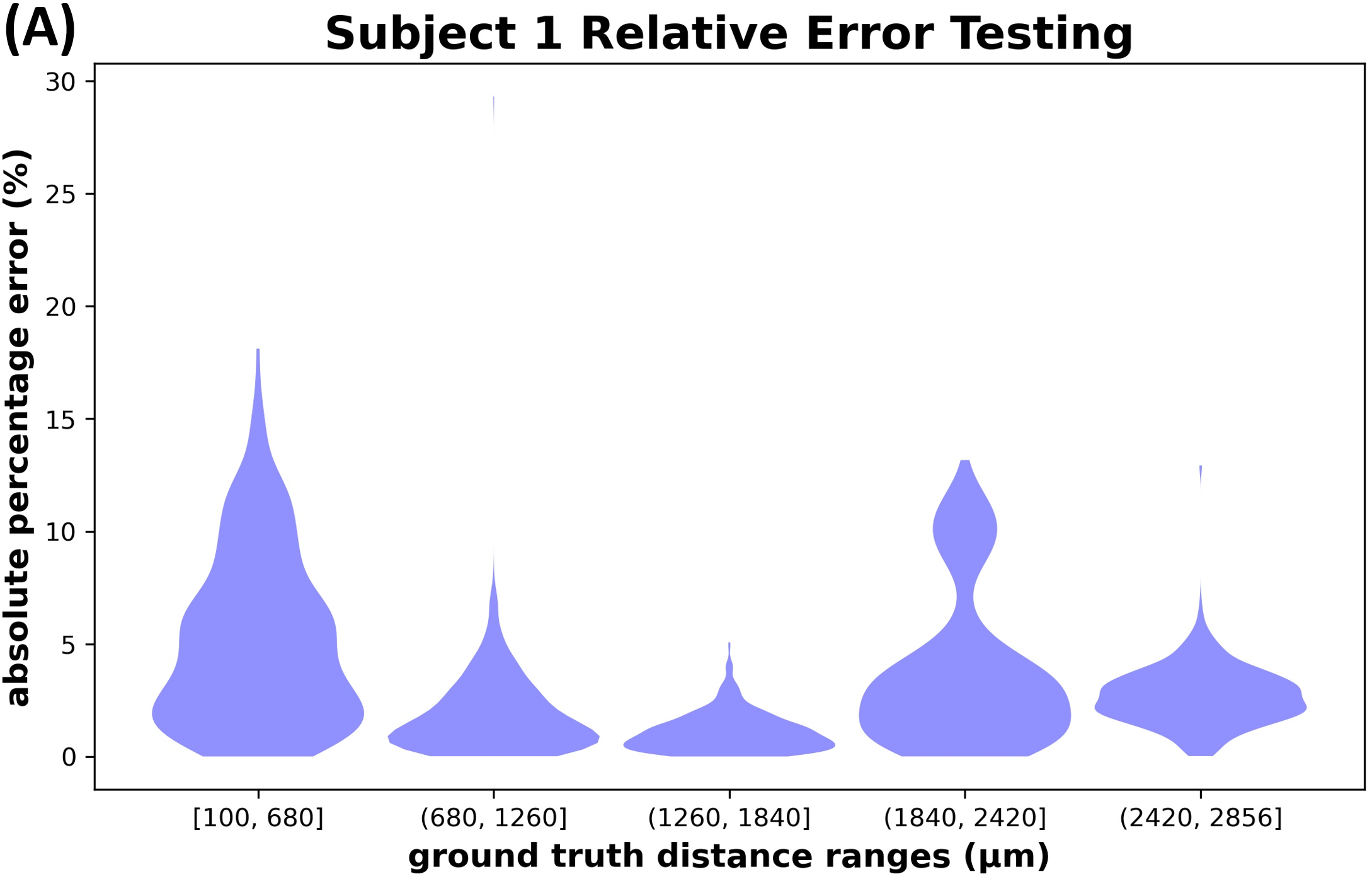

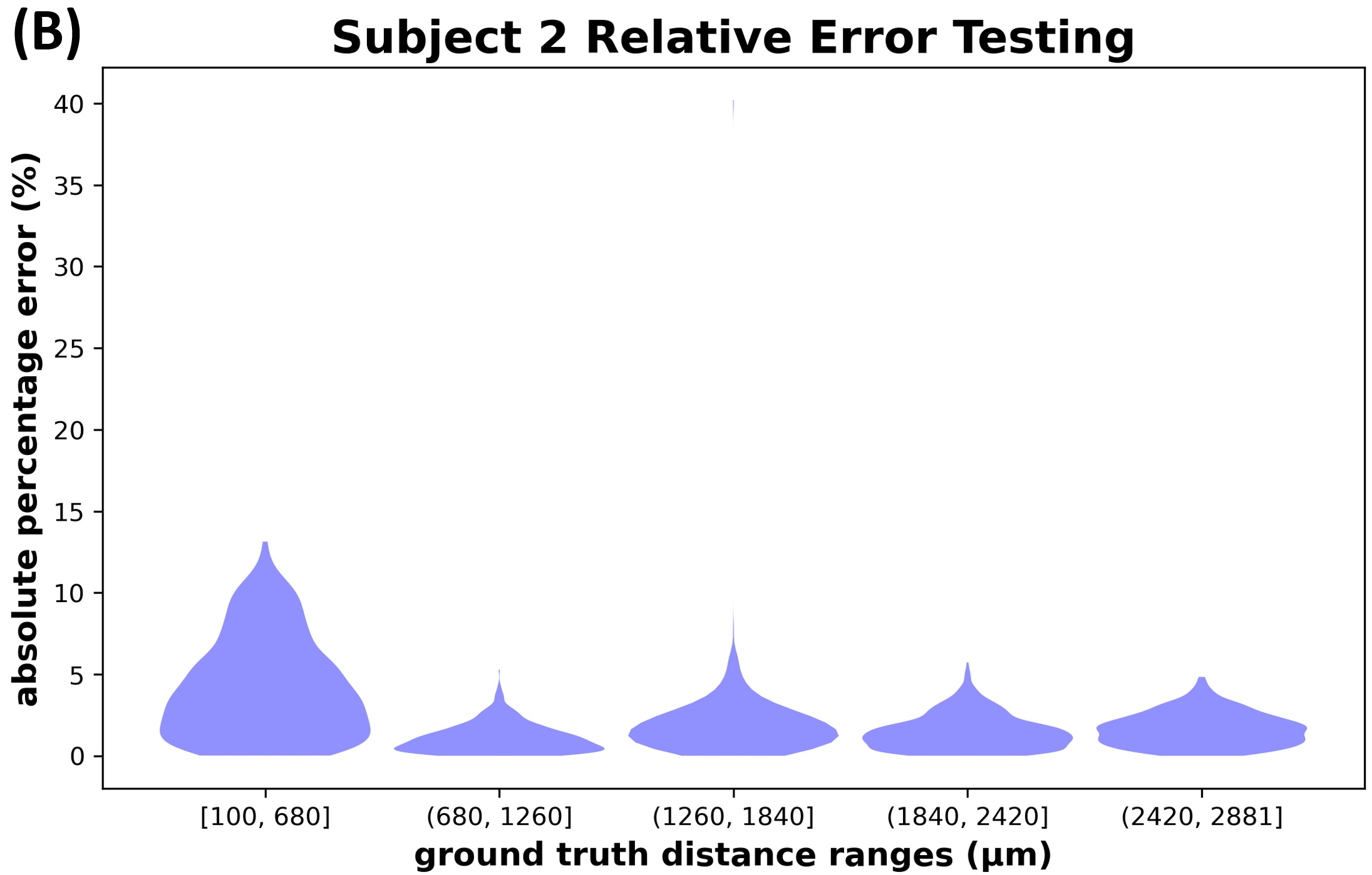


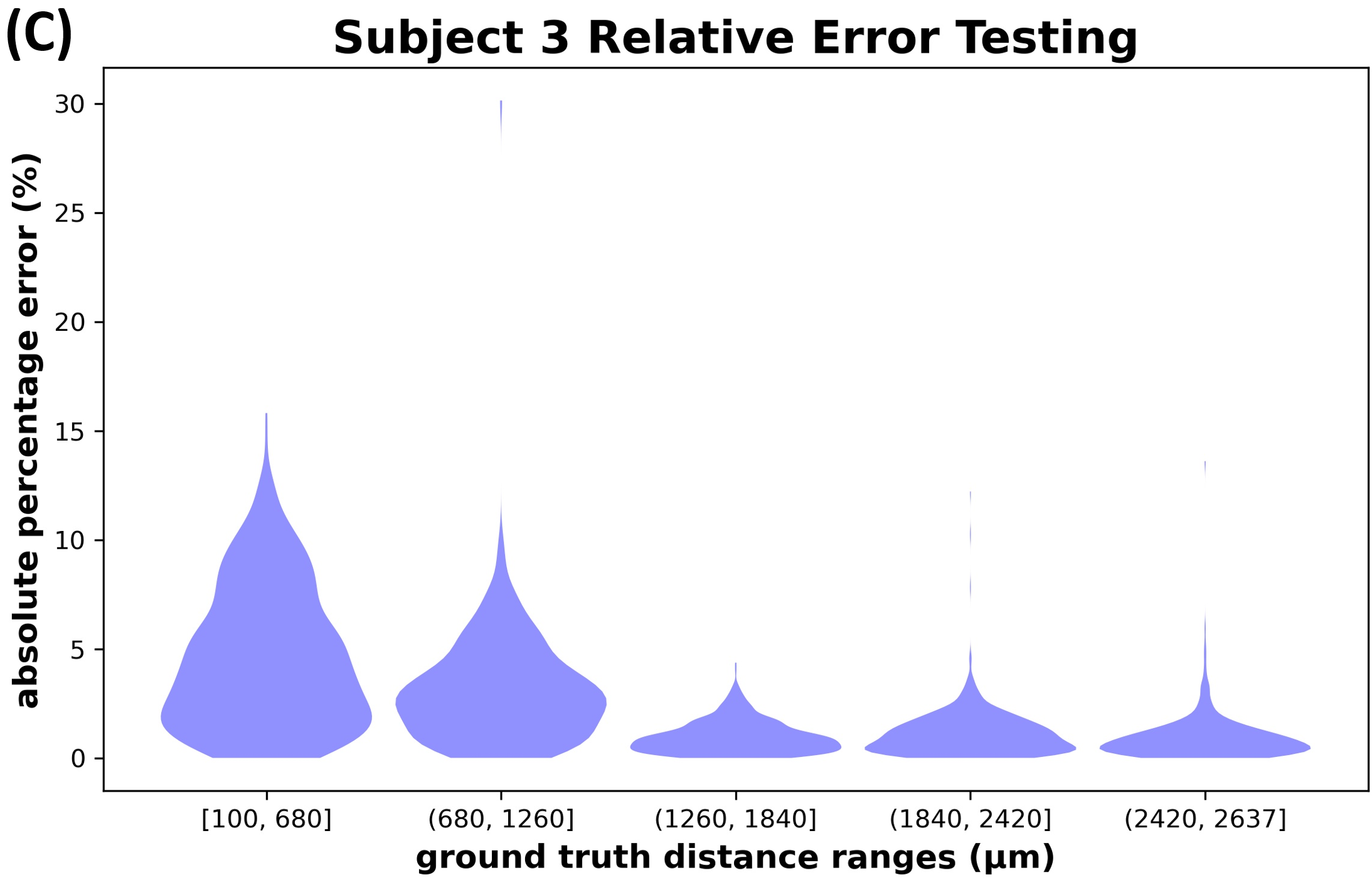

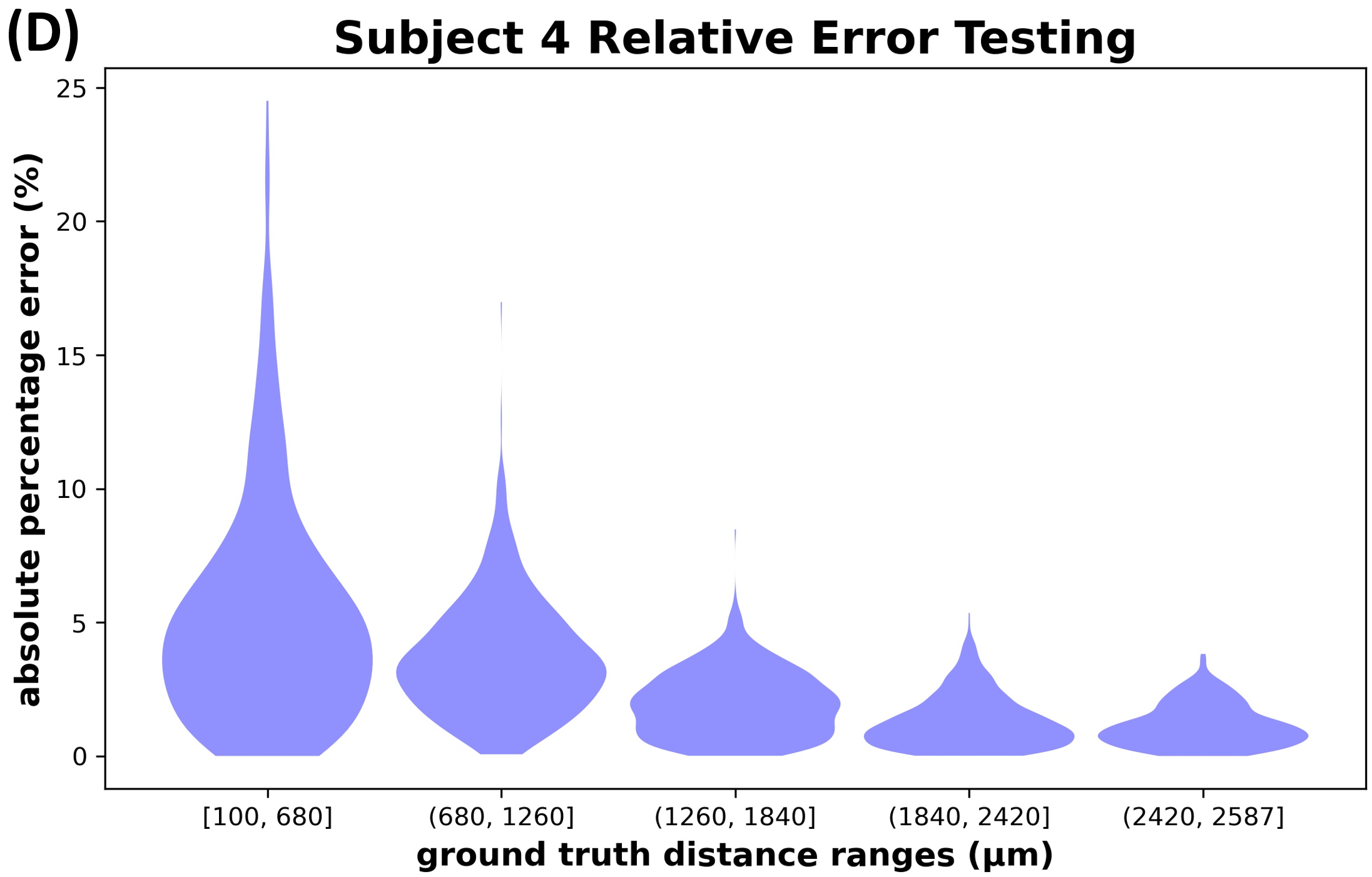


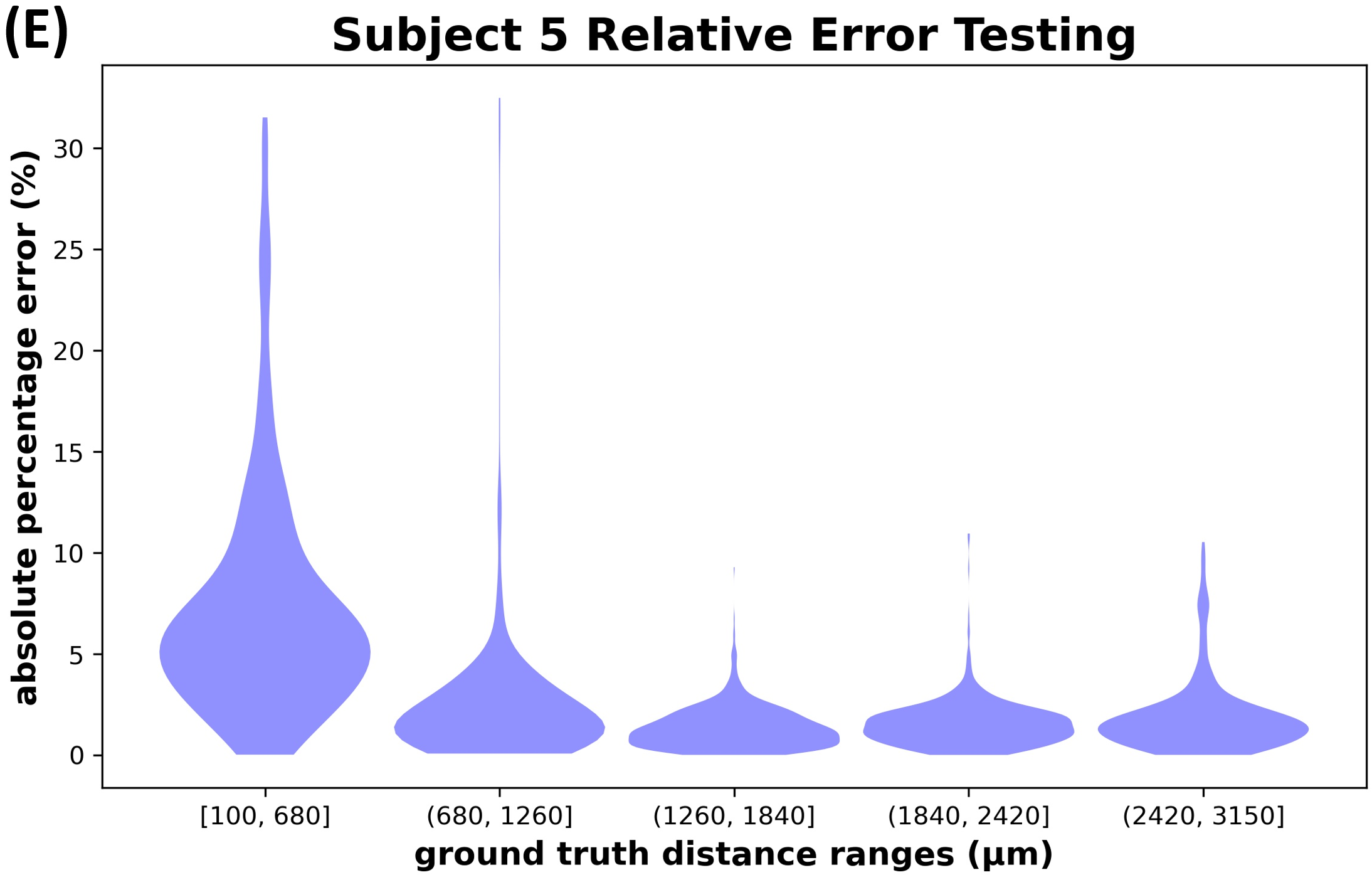

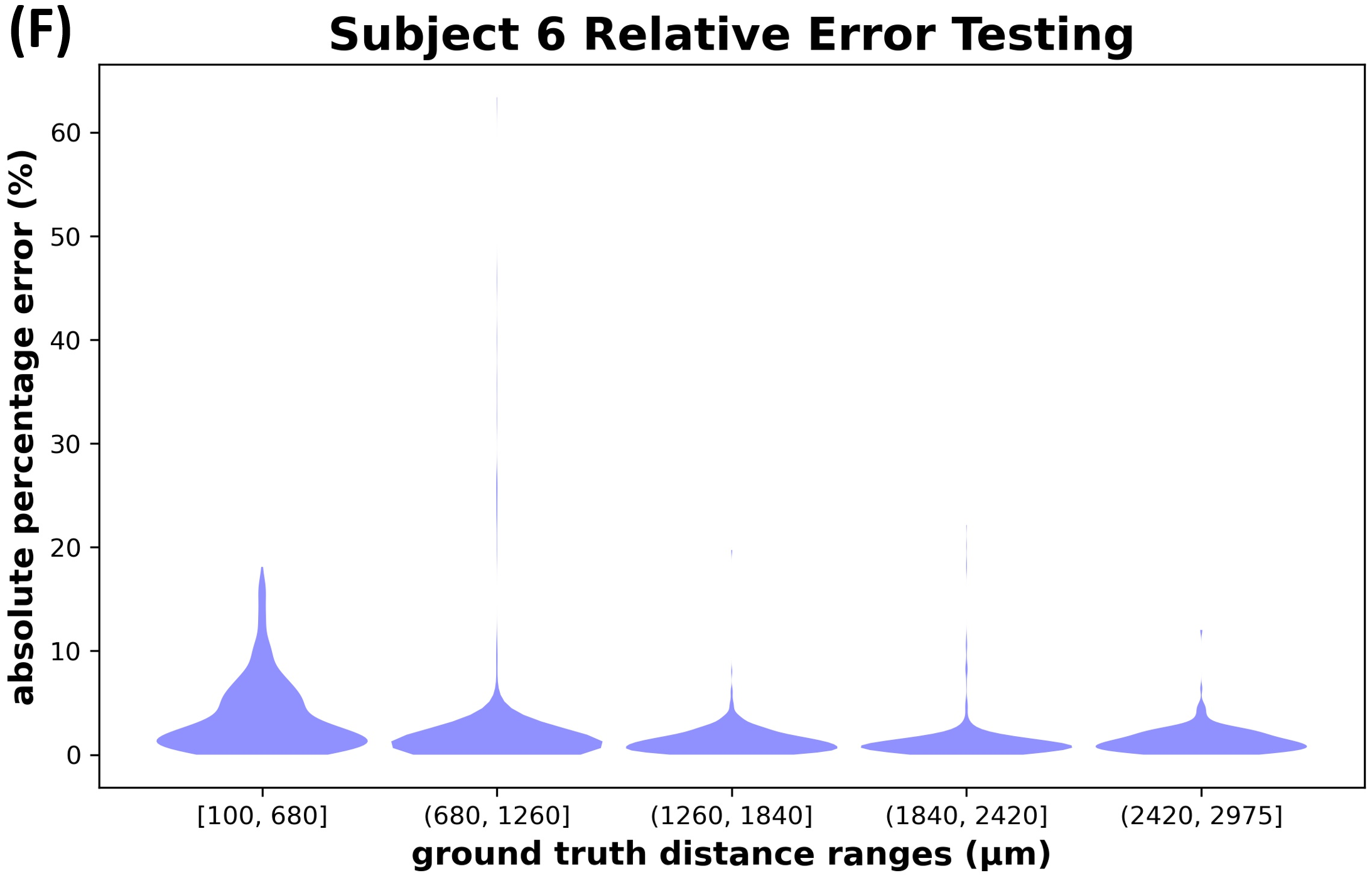


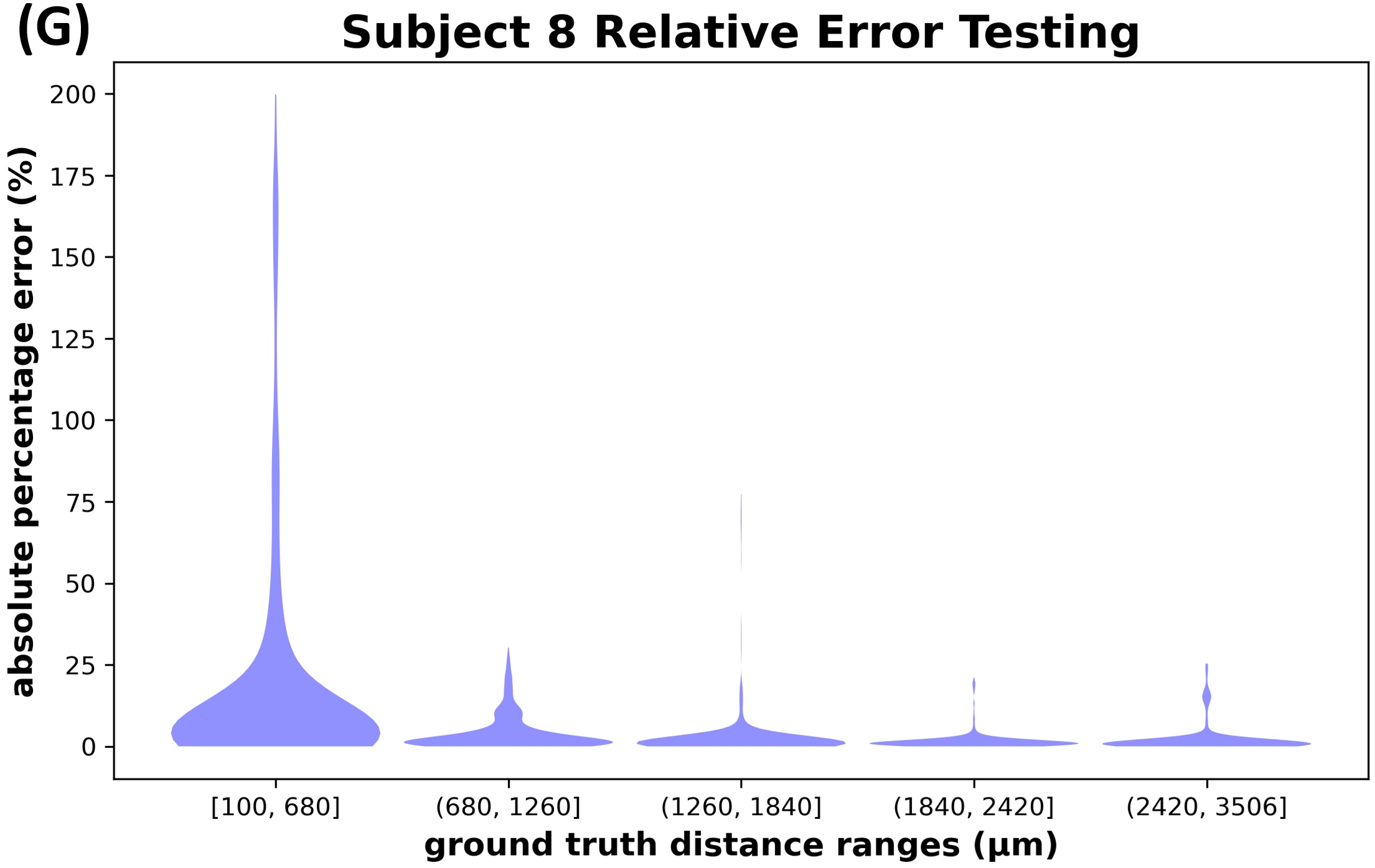


**Fig. 5.** Violin plots of the relative percentage error for the predicted distances.


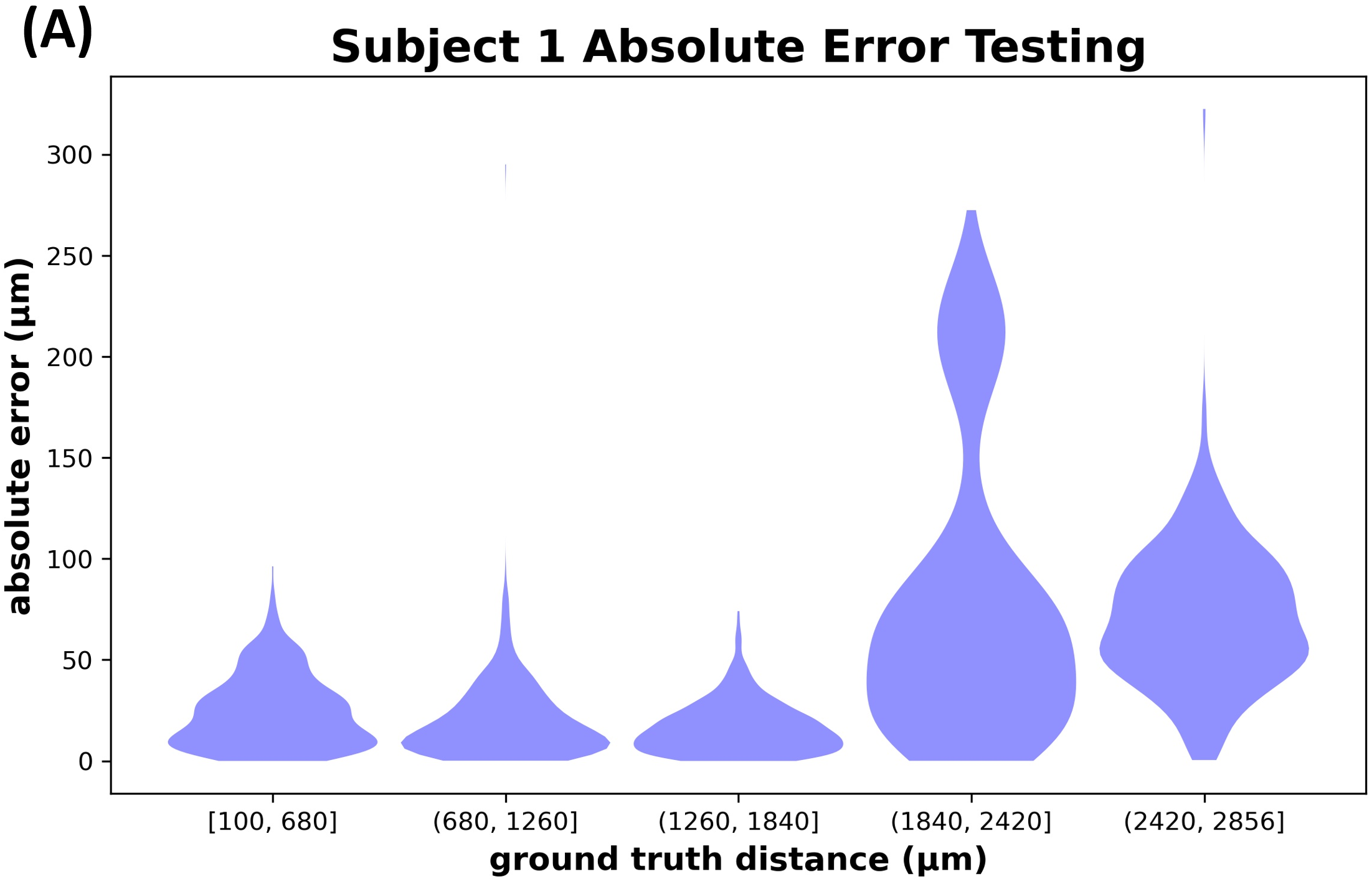

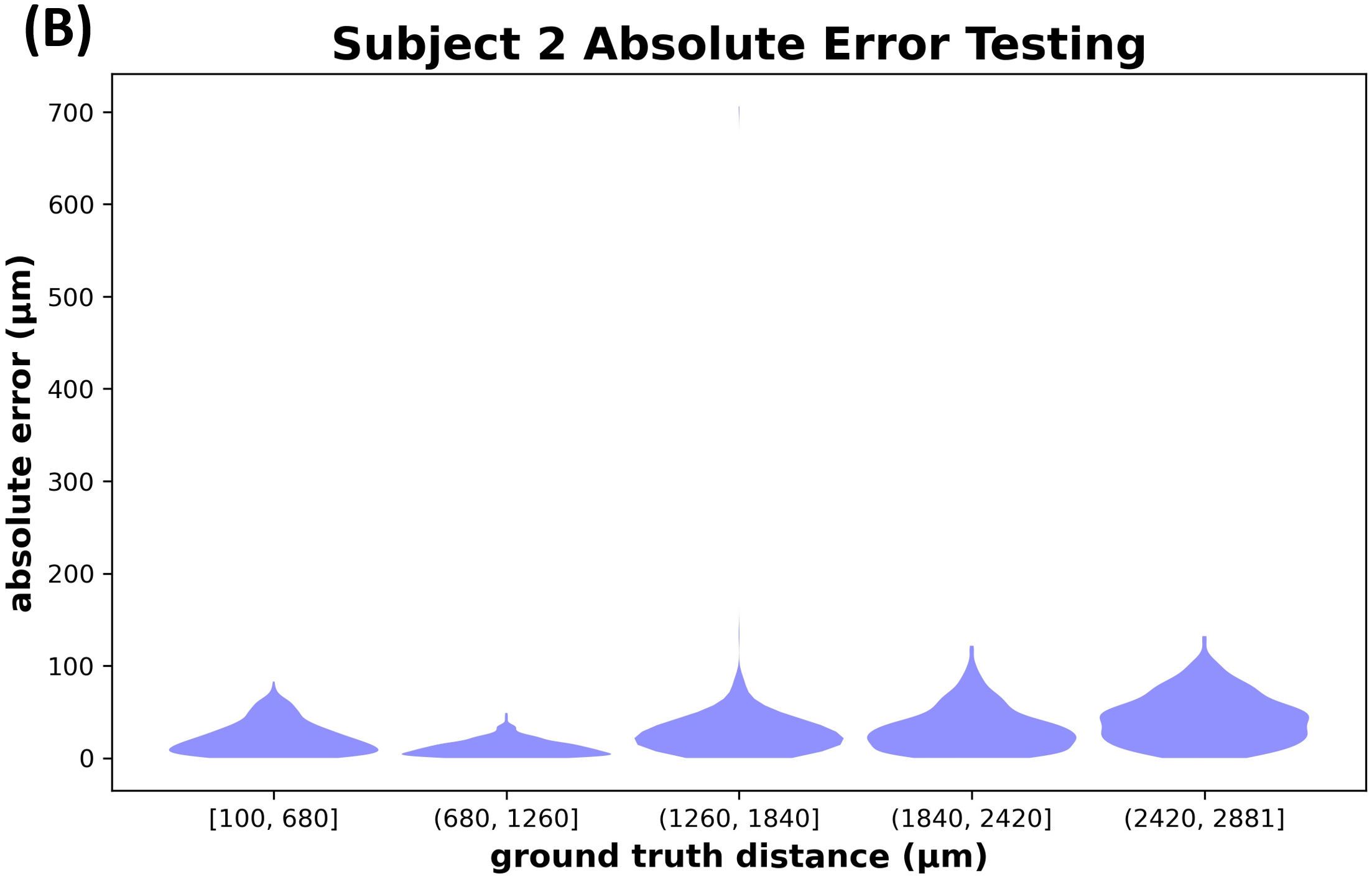


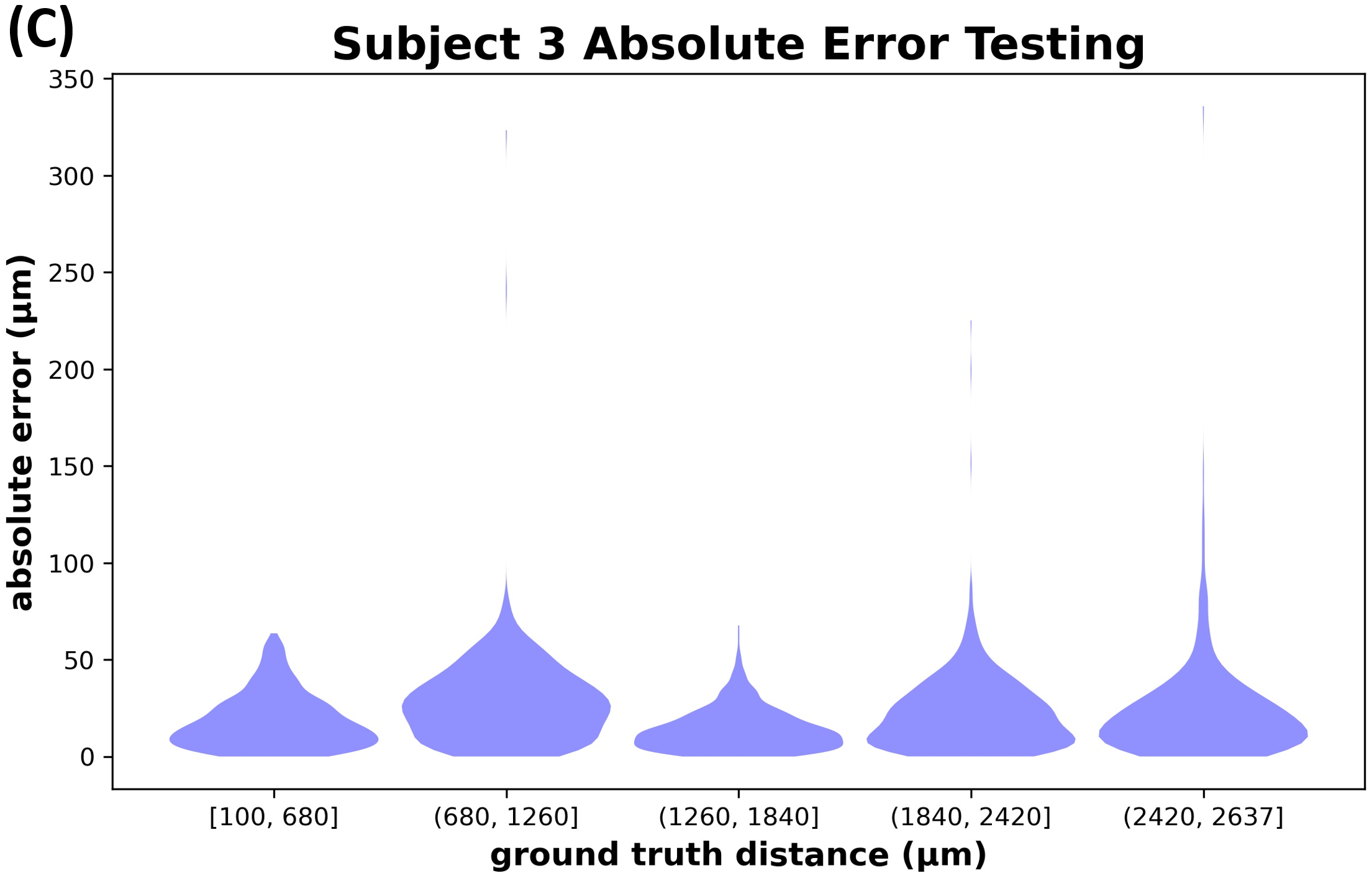

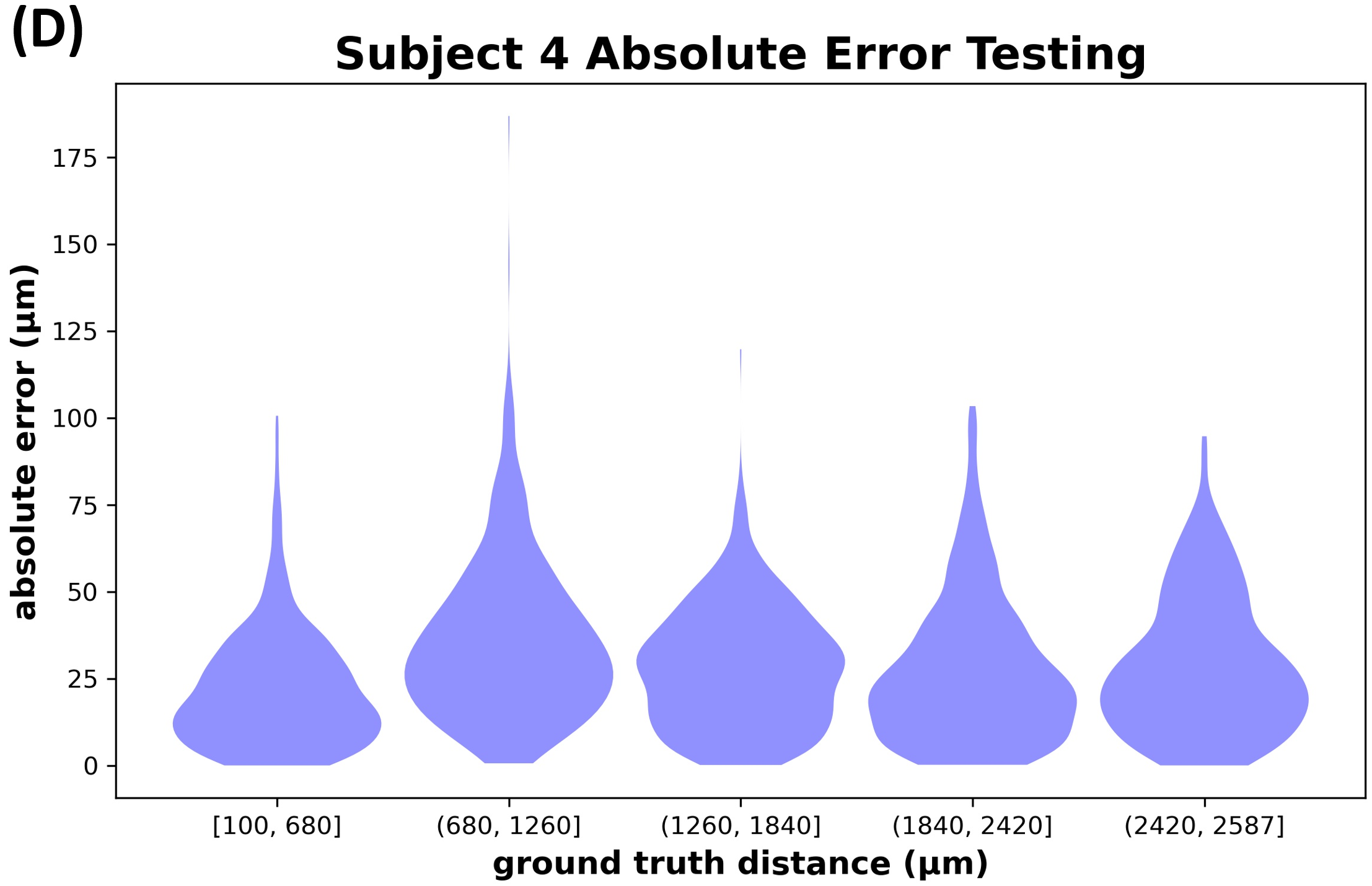


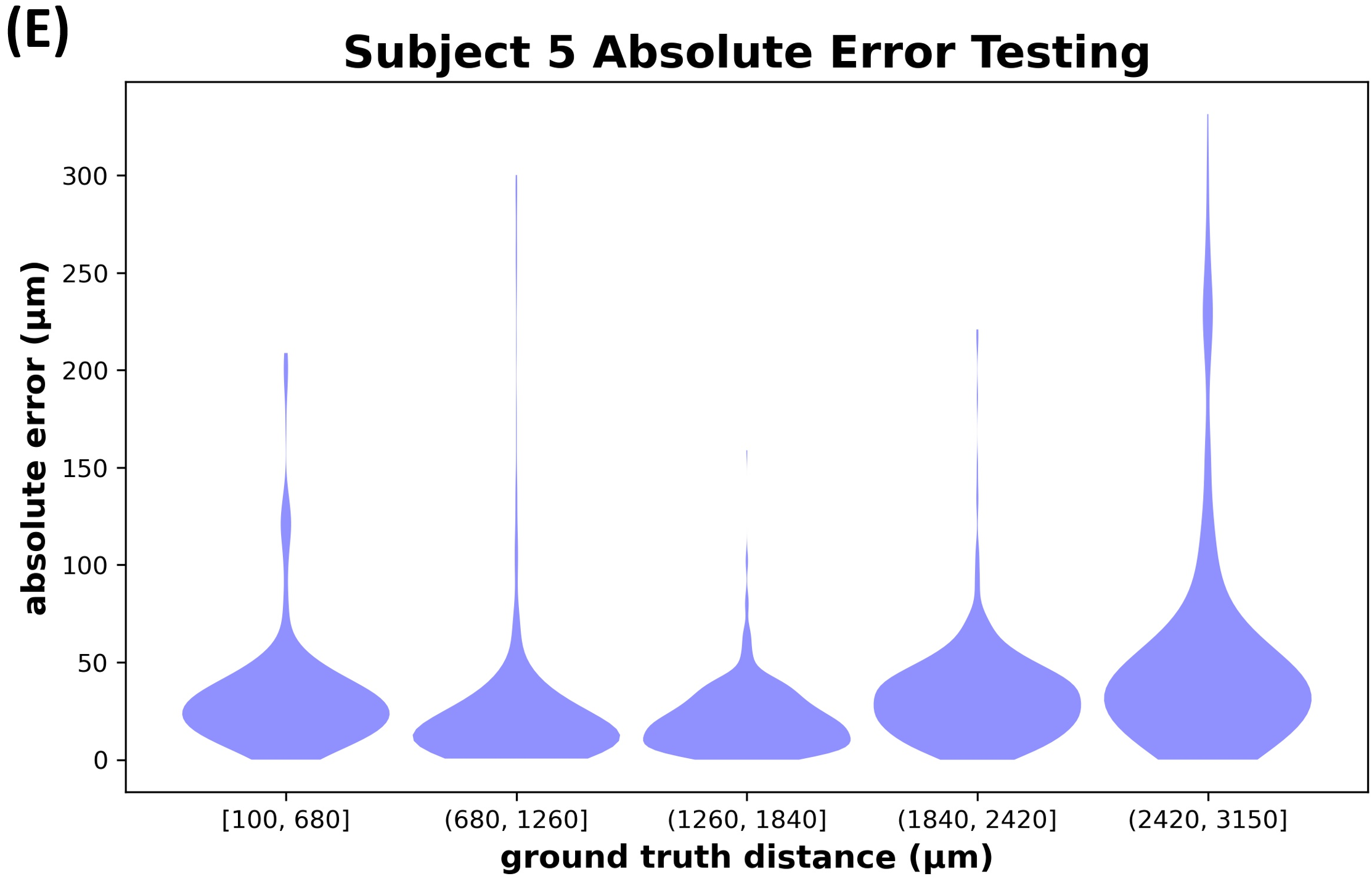

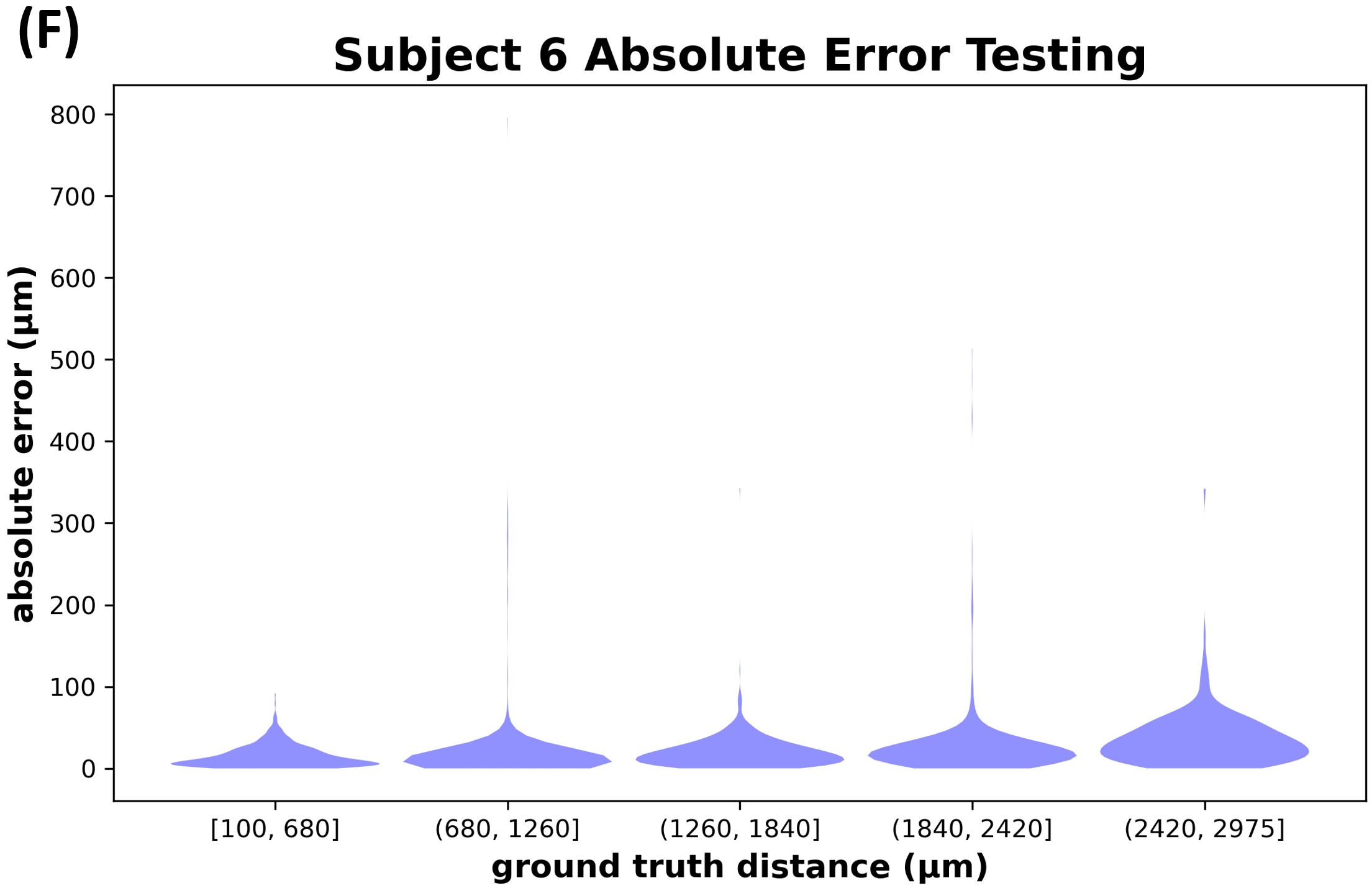


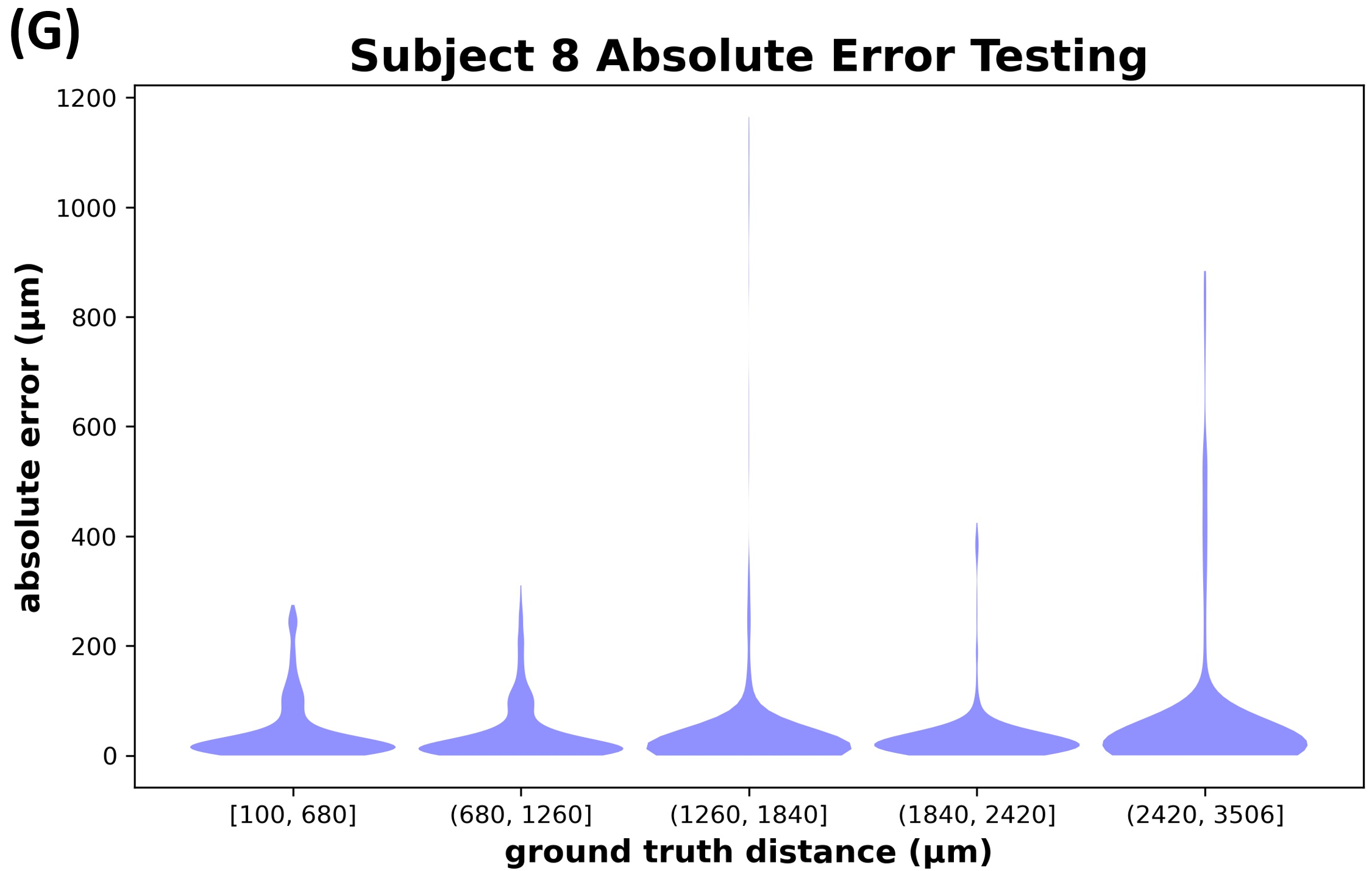


**Fig. 6.** Violin plot of the absolute error for the predicted distances.


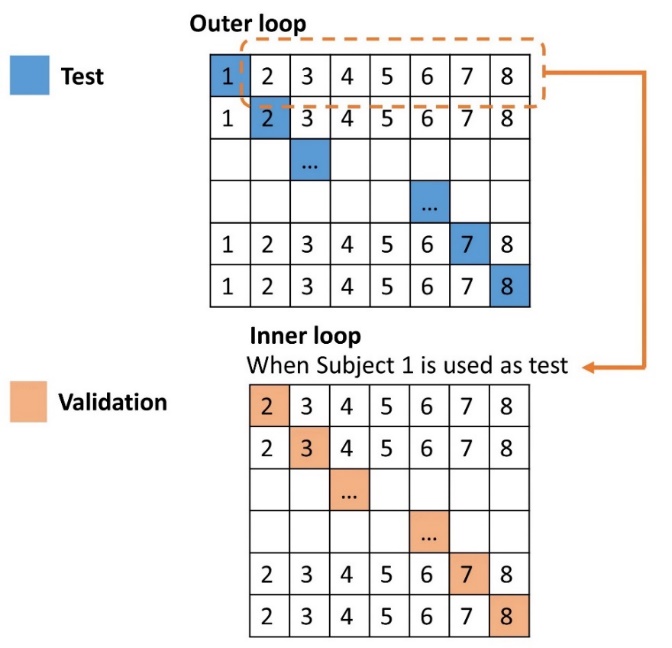


**Fig. 7.** Nested cross-validation and cross-testing. The example shows the first fold of the cross-testing loop with the images from Subject 1 held out for testing and the remaining images used for cross-validation. The cross-validation loop compares the average performance of three CNN architectures in each of Subject 2 to 8. The partitioning of images by subjects in the cross-testing and cross-validation allowed evaluation of the models' generalization accuracy across subjects.
